# Bioactive Glass and Silicate-Based Ceramic Coatings on Metallic Implants: Open Challenge or Outdated Topic?

**DOI:** 10.3390/ma12182929

**Published:** 2019-09-10

**Authors:** Giulia Brunello, Hamada Elsayed, Lisa Biasetto

**Affiliations:** 1 Department of Management and Engineering, University of Padova, Stradella San Nicola 3, 36100 Vicenza, Italy; 2 Department of Neurosciences, Section of Dentistry, University of Padova, Via Giustiniani 2, 35128 Padova, Italy; 3 Department of Industrial Engineering, University of Padova, Via F. Marzolo 9, 35131 Padova, Italy or; 4 Ceramics Department, National Research Centre, El-Bohous Street, Cairo 12622, Egypt

**Keywords:** bioactive glass, bioactive silicate ceramic, coating, implants, osseointegration

## Abstract

The overall success and long-term life of the medical implants are decisively based on the convenient osseointegration at the hosting tissue-implant interface. Therefore, various surface modifications and different coating approaches have been utilized to the implants to enhance the bone formation and speed up the interaction with the surrounding hosting tissues, thereby enabling the successful fixation of implants. In this review, we will briefly present the main metallic implants and discuss their biocompatibility and osseointegration ability depending on their chemical and mechanical properties. In addition, as the main goal of this review, we explore the main properties of bioactive glasses and silica-based ceramics that are used as coating materials for both orthopedic and dental implants. The current review provides an overview of these bioactive coatings, with a particular emphasis on deposition methods, coating adhesion to the substrates and apatite formation ability tested by immersion in Simulated Body Fluid (SBF). *In vitro* and *in vivo* performances in terms of biocompatibility, biodegradability and improved osseointegration are examined as well.

## 1. Introduction

Ceramics represent the class of materials that are more similar to bone in terms of composition, however, their intrinsic brittleness makes them unreliable for load bearing applications. For this reason, metallic materials are—thanks to their intrinsic ductility—commonly used for the production of orthopedic and dental implants. However, these materials have shown many disadvantages, including the release of toxic ions and the lack of integration at the interface between the bone and the biomaterial [1,2,3].

Titanium (Ti) and titanium alloys are widely used for the production of both orthopedic and dental implants because of their desirable mechanical features, such as relatively low modulus and good fatigue strength, their corrosion resistance and biocompatibility [4]. The α commercially pure Ti (CpTi) and the α-β Ti-6Al-4V alloy remain the most widely used materials for the intended purposes. Despite the use of stainless steel, in particular of AISI 316L (316L SS), remains widespread due to good mechanical properties and low cost, there are many concerns regarding the release of metal ions from the implant because of corrosion and wear [5]. Compared to stainless steel and cobalt-chromium alloys, Ti and its alloys have shown superior mechanical and biological properties [6,7,8].

Beside non-resorbable metals, magnesium (Mg) and Mg alloys are being employed as well in orthopedics [9,10,11], but due to their rapid biodegradability [12] they are not good candidates for dental implant applications.

Osseointegration has been considered as a key factor for the long-term success of biomedical implants. In order to obtain improved osseointegration and to shorten the time for osseointegration, enhancing implant stability in the early phases, several implant surface modifications have been explored [13,14].

For instance, the surface roughness has been demonstrated to affect the bone-implant interactions [7], and for this reason several studies have attempted to develop modified surfaces by means of physical and chemical approaches (e.g., sandblasting, acid etching, combination of blasting and etching, electrochemical oxidation and laser treatments).

**Figure 1:**
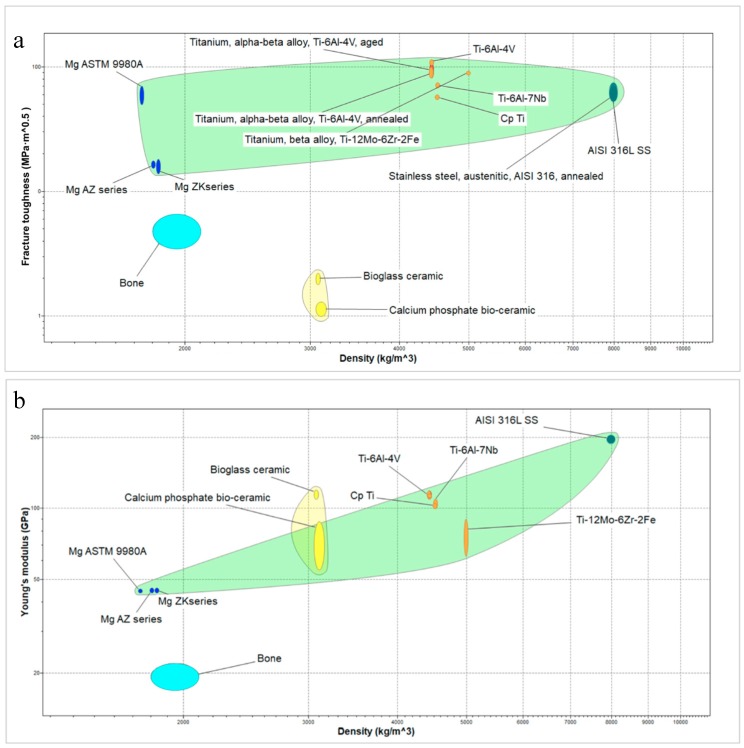
(**a**) Fracture toughness and density; (**b**) elastic modulus and density of bone, coating materials and most common metallic materials used in orthopedics and implant dentistry. Data elaborated using CES EduPack™ 2018.

Coating materials can be employed as well to further modify the implant surfaces in order to improve the performances of metallic implants. Material surface features play a crucial role in the chemical and biological interaction with the surrounding bone tissue, while the mechanical properties are strongly determined by the bulk of the implant [7]. Poor mechanical properties of monolithic bioceramics and bioactive glasses limit their use in load-bearing applications (see Figure 1a). As a consequence, the materials of choice still remain metallic alloys, whose biological properties can be improved by means of coatings (e.g., bioactivity, reduction of corrosion and toxic ion release) [1,9,15,16,17].

Metallic biomaterials currently used in orthopedics have a higher elastic modulus (in the range between 44 and 205 GPa) as compared to that of natural bone (17–22 GPa) (see Figure 1b), resulting in stress shielding effect that leads to a reduced stimulation of bone formation and remodeling and, subsequently, to the loosening of the implants [18,19,20,21]. It means that the implants “shield” the surrounding bone from experiencing adequate loading, which is required for bone growth stimulation, and when the stress level decreases too much, bone resorption may occur. Low elastic modulus, better matching that of bone, reduces the stress-shielding effect, thus favoring bone formation and improving implant osseointegration. The elastic modulus of magnesium is closer to that of natural bone, but its rapid degradability hampers its clinical application [9]. The long-term behavior of implants represents one of the most important concerns, where fatigue fracture is one of the main causes of failure. Indeed, it was recently observed that the bone-implant difference of elastic modulus not only affects the bone to implant penetration but the long term behavior of the implant itself as well: the closer the elastic moduli are, the smoother is the bone- to implant stress transmission, thus improving the long-term mechanical behavior of the implant itself [22]. Titanium alloys, such as α-β alloys show an improved fatigue behavior, compared to pure CpTi, but the dissolution of alloys elements may be toxic for the growing bone [23]. For this reason, a novel generation of β-stabilized alloys have been developed (such as Ti-29Nb-13Ta-4.6Zr) showing a Young module close to that of bone and improved corrosion resistance [24]. Implant geometry can prevent stress-shielding, as described, for instance, in a prospective study evaluating total hip arthroplasties, in which a modified femoral stem design led to a significant improvement in stress-shielding in the proximal femoral bone [25]. Apart from implant macro design and from the development of a porous structure, decreasing the overall stiffness of the prostheses [26] is a way to mitigate the effect of stress-shielding, consisting in improving bone-implant bonding using bioactive coating materials [27,28]. While the stress shielding effect has been deeply investigated and recognized in the field of long bone researched, it is still controversial its role in implant dentistry [29,30].

Among biomaterials used for coating metallic implants, hydroxyapatite (HA) is one of the most widely used and documented biomaterials [31,32]. It has been successfully applied—showing promising results for osseointegration—thanks to its resemblance to the inorganic compound of natural bone tissue [33,34]. Unfortunately, early experiences with HA coatings revealed frequent delamination of the HA coatings from the substrates and inadequate chemical stability, thus compromising the long-term success of the implants [7,35,36]. Over the last few years, to overcome the limitations of HA coatings, many attempts have been performed to develop chemical stable coatings showing good adhesion to the substrates and high bioactivity. It has been demonstrated that ceramic materials and bioactive glasses can stimulate bone formation both *in vitro* and *in vivo* [1,15,31,32,37,38,39]. Moreover, the use of coating materials as delivery systems—incorporating antibiotics and antimicrobial agents or bone stimulating molecules such as growth factors (GFs)—has been reported [40,41,42,43].

The goal of the present article is aimed at providing a literature review of most relevant and recent findings on the topic of bioactive glasses and bioactive silicate ceramics as coating materials for metallic biomedical implants, in order to clarify whether the coating approach represents an outdated method or an open challenge for improving implant performances. In this review, the main mechanical features will be introduced, with particular focus on coating roughness, thickness and adhesion to the substrates. Moreover, coating behavior *in vitro* and *in vivo* will be discussed.

## 2. Microstructural Features of Metallic Substrates

The material features of commonly used metallic substrates for implant applications will be discussed in the following paragraph. The main mechanical (Young’s modulus; Vickers hardness), thermal (coefficient of thermal expansion; melting temperature) and physical (density) properties of these metallic materials are summarized in Table 1 and compared to those of bone tissue. 

### 2.1. Ti and Ti Alloys

Ti and some of its alloys, in particular Ti-6Al-4V, are largely used as implant materials both in orthopaedics and in dentistry due to their excellent biocompatibility, high corrosion resistance, and good mechanical properties [44,45,46]. As compared to CpTi, the α-β Ti-6Al-4V alloy exhibits higher strength, maintaining similar biocompatibility [47]. Main mechanical and thermal properties of Ti and Ti-6Al-4V are summarized in the Table 1.

Ti alloys can be classified based on the presence and proportion of α- and β-phases. Alloying elements such as aluminum (Al), carbon (C), oxygen (O) and nitrogen (N) are commonly used to stabilize the α-phase, while elements such as molybdenum (Mo), vanadium (V), niobium (Nb), iron (Fe), manganese (Mn) and chromium (Cr) are considered β-stabilizers [4,48].

Despite their favorable performances for hard tissue replacements, there are still some concerns with the use of many Ti alloys due to the release of cytotoxic or allergic elements such as Al [49,50]. This release could be caused by corrosion processes, wear or a synergistic combination of both, known as tribocorrosion [49,51]. Elements released into the surrounding tissues may affect peri-implant cell behavior and represent a potential risk for the long-term success of the implants [49,52].

Furthermore, Ti and its alloys seem to insufficiently osseointegrate at the early stage after placement due to their moderate osteoconductivity [53]. As highlighted in a recent review on modern implant surfaces for dental applications [46], there is a high demand for new surfaces able to induce a faster osseointegration and to increase the predictability and success rate of implant treatments, as well as in areas with low bone quality or in systemically compromised patients in which bone healing might be impaired. To this aim, several surface modifications of Ti and Ti-alloy implants have been proposed, by means of subtractive processes, additive processes or a combination of both [44,46]. Subtractive treatments include grinding, polishing, blasting, acid-etching and laser texturing, whereas additive surface treatments mainly consist in coating depositions by means of different methods, such as plasma-spraying or electrophoretic deposition. Finally, biofunctionalization of surfaces with peptides, growth factors or drugs, has been proposed as an alternative promising strategy for improving the implant osseointegration [54,55,56].

### 2.2. Stainless Steel 

Stainless steel implants have been widely employed in orthopedics, as temporary fixation devices as well as fixed implants for joint replacements [5]. As compared to other biomaterials, such as Ti, stainless steels are considered inferior in terms of biocompatibility and resistance to corrosion. However, stainless steel is cheaper and suitable for the fabrication of orthopedic implants. Due to economic issues, there is still a high demand for stainless steel implants—in particular in developing countries where an affordable healthcare has to be guaranteed to the population [57].

Stainless steels are generally well tolerated by the human body, due to the development of an outer chromium-rich oxide passive layer on the surface. Stainless steels present three main microstructures: ferritic, austenitic and martensitic. On one hand, only ferritic stainless steels do not contain nickel (Ni), whereas only austenitic stainless steels are non-magnetic. Hence, regardless of the presence of Ni, austenitic stainless steels have mainly been used in orthopedics because the absence of ferromagnetism is required. In particular, the most extensively used for biomedical applications is the austenitic stainless steel (AISI 316L, ASTM F-55 and F-138), containing Cr (17% to 20%), Ni (12% to 15%), Mo (2% to 3%) and small quantity of other elements [5,58]. This material, similar to the other austenitic stainless steels, is characterized by a face-centered cubic (fcc) structure and satisfactory corrosion resistance. Moreover, it possesses acceptable biocompatibility and good mechanical properties also for load-bearing purposes [5].

The major disadvantage of stainless steels is the high elastic modulus as compared to that of natural bone, which may lead to stress shielding. In addition, there are many concerns about the increased risk of localized and systemic reactions associated with the release of metals into the human body—especially of nickel [59]. For instance, allergic reactions are commonly reported when Ni-containing stainless-steel devices are implanted.

In order to overcome the adverse effects associated with the release of nickel, nickel-free austenitic stainless steel has been proposed [60]. Among elements which are used in order to stabilize the austenitic microstructure (i.e., nickel, cobalt, carbon, nitrogen, manganese, and copper), nitrogen seems to be the most promising nickel replacement, because it is a strong austenite forming element and it improves the mechanical properties and the corrosion resistance of the steel as well [5,60,61].

The improvement of implant properties can be achieved either by modifying the chemical composition of the stainless steels, such as eliminating Ni content, or by applying a bioactive coating on the surface [5,57].

Coating materials applied onto stainless steel for biomedical purposes have been investigated as well.

Tribological stability and reduced amount of metal debris released from the implants have been documented when bioactive coatings have been deposited [57,62,63].

### 2.3. Mg and Mg Alloys

In recent years, there has been an increasing interest in Mg and its alloys as orthopedic implant materials, due to their attractive properties, such as biocompatibility and biodegradability [64,65,66].

The elastic modulus of Mg and Mg alloys is quite similar to that of natural bone. This prevents the occurrence of the “stress-shielding” phenomenon, which is one of the foremost causes of orthopedic implant failures [64,65].

The biodegradability of Mg and Mg alloys can be considered a favorable characteristic, as a second surgery for the removal of the implant in certain orthopedic applications becomes unnecessary [67]. Despite the beneficial properties of Mg and its alloys, their degradation rate is too fast, as compared to new bone formation and remodeling around the implants and may lead to an early loss of the mechanical integrity of the bioresorbable implants [67]. Therefore, the application of these materials is currently limited to non-load bearing conditions in most cases.

Although the release of Mg ions is not related to any severe side effect in the human body [68], the rapid release of hydrogen due to the corrosion of the implant is an important issue during the healing process. When the release of gas is too quick, the gas cannot be absorbed by the body in time, producing the well-known “balloon effect” [69]. However, even if a considerable percentage of patients experienced subcutaneous gas cavities, most of them had no pain or infections after surgery [70].

Many strategies have been developed in order to control the degradation rate and improve the corrosion resistance of Mg implants, including alloying [71], thermal treatment [72] and coating deposition [11].

Several alloying elements, including Al, calcium (Ca), zinc (Zn), zirconium (Zr), strontium (Sr), Mn, etc., have been employed to enhance the properties of biodegradable Mg implants. Intermetallic phases, resulting from the reaction of alloying elements among each other or with Mg, can be found along the grain boundaries or dissolve within the Mg matrix, modifying the mechanical behavior and the corrosion resistance of the material [71]. The general trend is that alloying elements decrease the corrosion rate. However, it is not possible here to quantify their effect in terms of corrosion resistance, since data in literature are not homogeneous [73,74].

The release of metal ions, commonly used as alloying elements, from Mg-based orthopedic implants may give rise to systemic or localized adverse reactions. For instance, an excess amount of Al, which is a constituent of many alloys used in biomedical devices (e.g., AZ31, AZ91 Mg alloys), may lead to neurological disorders or a decrease in osteoclast viability. However, the low concentration of toxic elements is generally well-tolerated by the body [74].

The deposition of bioactive coatings seems to be the most attractive method to reduce the degradation rate and, in the meantime, to improve the biocompatibility and the medium-term mechanical integrity of the both Mg and Mg alloy implants [66].

## 3. Microstructure, Physical Features and Applications of Bioactive Glasses and Silica-Based Bioceramics

### 3.1. Bioactive Glasses

The first composition, the so-called 45S5 Bioglass^®^ (in wt.%: 45% SiO_2_, 24.5% CaO, 24.5% Na_2_O, 6.0% P_2_O_5_), was discovered by Hench in the 1960s. It has been in the clinical use since the 1980s and it remained the most used bioactive glass [75]. 

The bioactivity of this material is associated with the ability to induce the formation of an apatite layer on its surface upon immersion in simulated body fluid (SBF). It has been extensively demonstrated that bioactive glasses possess high bioactivity [76,77]. Moreover, bioactive glass-ceramic substrates were found to increase osteoblast differentiation and selection of a mature osteoblastic phenotype *in vitro* [78].

With regard to the clinical applications, 45S5 Bioglass has been successfully introduced in alveolar ridge maintenance [79], middle ear replacement [80] and periodontal surgeries [81,82]. 45S5 Bioglass has been successfully incorporated in toothpastes as an active repair agent, able to mineralize dentin and thus reducing dental hypersensitivity [83]. It has been proposed as bone substitute material for the treatment of complex bone defects as well. However, the low mechanical strength and the high brittleness of porous glass scaffolds have limited their application to repair of non-load-bearing bone defects [84]. Interestingly, in particular for bone tissue regeneration purposes, various attempts have been made to regulate the degradation rate and to improve the mechanical strength of bioactive glasses by modifying their chemical composition by means of the incorporation of metal oxides (e.g., MgO, ZnO, B_2_O_3_, Al_2_O_3_) [85]. Bioactive glasses have been proposed as coating materials for implant devices as well. A promising approach for increasing the implant bioactivity consists in the deposition of bioactive glasses on bioinert metallic substrates [86]. This allows, on one hand, to improve the biological properties of the metallic implants and, on the other hand, to overcome the major drawbacks of the glassy phase—which are their poor mechanical properties and their brittle behavior.

More recently, besides the original 45S5 Bioglass, a broad variety of bioactive glasses with different chemical compositions have been developed [87]. They contain SiO_2_, Na_2_O, CaO, and P_2_O_5_ in different ratio (Table 2). In Finland a bioactive glass known as S53P4, containing a higher percentage of silica as compared to 45S5 Bioglass was successfully developed, with encouraging results in the treatment of bone defects [88,89,90]. Glass compounds containing other elements as well have been developed, such as 13-93 glass. Both S53P4 and 13-93 bioactive glasses were tested in a frontal sinus and skull bone defect obliteration model *in vivo*, showing enhanced bone healing as compared to synthetic HA [82,91]. In addition, a faster bone recovery was observed in defects treated with S53P4 than in ones filled with 13–93. This might be related to the presence of Mg in 13-93 glass, reducing its bioactivity. Other bioactive compounds are based on CaO-SiO_2_ combinations, like 70S30C, 58S and 77S bioactive glasses [87,92].

Additionally, in order to improve the mechanical properties of bioactive glasses, a new class of materials—known as bioactive glass-ceramics—was developed, starting from several glasses undergoing the precipitation of crystalline phases during heat treatment [93,94,95].

### 3.2. Silica-Based Ceramics

Bioceramics are commonly used in several applications in medicine, included orthopedics and dentistry [100]. As a new family of bioceramics, silica-based ceramics have been afforded a considerable amount of importance as coating materials on the surface of biomedical implants, as well as bone substitutes [101,102,103].

The main silica-based ceramics used for medical devices are reported in Table 3. 

There is an increasing evidence that silicon (Si) can promote the new bone formation [104], as confirmed by both *in vitro* and *in vivo* studies. Contrary to conventional calcium phosphate ceramics, such as hydroxyapatite or β-tricalcium phosphate (β-TCP), the presence of Si in the composition of silica-based ceramics contributes to their bioactive properties. Moreover, owing to the wide range of chemical compositions, it is possible to tailor formulations of silica-based ceramics in order to meet specific application requirements in terms of mechanical properties, bioactivity and degradation rate [101,105,106,107]. For instance, the incorporation of trace elements, such Mg or zinc Zn, into Ca-Si ceramics has a considerable influence on the mechanical properties of ceramics, both on bending strength and on fracture toughness. In particular, bulk silicate ceramics with MgO–CaO–SiO_2_ (e.g., akermanite, bredigite, diopside, merwinite) or ZnO–CaO–SiO_2_ (e.g, hardystonite) ternary oxides presented fracture toughness values in the range of 1.24–3.5 MPam^1/2^—higher than those of CaSiO_3_ and hydroxyapatite (≤1 MPam^1/2^) [105].

In addition, it is worth noting that most bioactive glasses and silica-based ceramics possess elastic modulus similar to that of human bone (Table 2 and Table 3), making them good candidates for orthopedic implants.

## 4. Deposition Methods and Physical Properties of the Coatings

Bioactive glasses and silica-based ceramics have been investigated as coating materials for both resorbable and non-resorbable metallic substrates, using a variety of synthesis and deposition methods. Superficial features—in particular the roughness and the thickness of the coatings—are of great importance for the characterization of the devices prior to other *in vitro* biological or *in vivo* test—these properties have been reported in Table 4 as well. 

Even though the surface roughness-osseointegration relationship is well-known, there are however still unclear aspects, as reported by Wennerberg and Albrektsson [130] regarding implant dentistry. For instance, the key morphological parameters are not standardized, hence, it is tough making comparisons between different studies. In this review, we are comparing the average roughness profile (Ra), as it is the most frequently used parameter in the investigated articles.

Coating thickness has been reported as well, because it strongly affects the dissolution time in body fluids. In addition, when coatings undergo a thermal treatment after deposition, the higher the coating thickness the higher are the induced thermal stresses, with consequences on adhesion strength to the substrate, so as on the mechanical integrity of the coating itself (i.e., cracks initiation) [131].

Even if porosity may be considered a key parameter for *in vitro* performances of the coating, we observed a lack of data in the literature. For this reason, these values could not be considered in the present review. 

The bioceramic coatings can be realized by means of production processes involving the synthesis and the deposition of the ceramics directly on the substrates (e.g., micro-arc oxidation or plasma spraying) [11,65,158].

Other processes comprise a first step, in which the precursors for glass or ceramic synthesis are for example mixed with a sol-gel technique [57,62,65,66,67,155,156,157] or from preceramic polymers [37,132,133,134], followed by a second step consisting in the deposition of a solution containing the precursors onto the substrates. When the deposition process occurs at high temperatures—as in the case of plasma [146] or thermal spaying [98]—no post-depositional heat treatment is required. Whereas when the deposition is carried out at room temperature (dip-coating, spin-coating or spray-coating), a heat treatment has to be applied to remove the solvent used in the preparation of the coating and the development of the desired bioactive coating.

Bioactive glass and glass-ceramic coatings are normally obtained by the melt-quenching route or by the sol-gel process [82,93] and are then applied onto the substrates in a second moment, followed by heat treatment. 

Several silica-based bioceramics were used to coat Ti and Ti-6Al-4V substrates, including sphene [37,127,132,133,134,135,136,137,138], hardystonite [135,139,140], Sr-substituted hardystonite [140], arkemanite [141], baghdadite [142], bredigite [143], diopside [123], dicalcium silicate [144] and wollastonite [53,145].

These coatings possess a Ra in the range of 0.4–12.1 μm. The great variability depends not only on the deposition method and on the roughness of the substrate, but on the dimension of the powders and on the synthesis technique as well. Rougher surfaces were obtained using plasma spraying, with Ra values above 5 μm. Ra below 5 μm has been found when other deposition methods have been applied (i.e., spray-coating and spin-coating).

Surface roughness of sphene ceramic coatings—prepared by the preceramic polymer processing route and deposited by spraying coating using an automated airbrush—varied with the composition of the solution and with the deposition time. The reduction of deposition time (1 s) and the use of nano-sized active precursors resulted in coating with Ra of 3.5 ± 0.5 μm [133]. Consistently, sphene-coated CpTi samples produced with the same method exhibited Ra and Sa of 3.9 ± 0.7 μm and 3.6 ± 0.5 μm, respectively [37]. However, lower values are reported in Biasetto et al. [134], whom used the same solution and deposition technique—however this might be related to the use of different Ti substrates.

Atomic force microscopy (AFM) analysis revealed a surface roughness of 0.38 ± 0.04 μm of sphene coatings synthesized via sol-gel and deposited by means of spin-coating [138]. The thickness of the silicate ceramic coatings on Ti-based substrates presented minimum values of 0.5 to 400 μm. The lowest values were observed when spin-coating was applied [138]. With this technique it is possible to deposit thin coating layers below 10 μm. Thicker coatings were produced with airbrushing deposition, with reported values around 100 to 150 μm [132,133,134]. Coating deposited using plasma spraying showed a broad variability, from 10 [136,140] to 400 μm [141,145].

Additionally, bioactive glass and glass-ceramics coatings on Ti-based substrates were investigated [1,86,98,146,147,148,149,150,151,152,153,154]. 

Surface roughness of these coatings is frequently not reported in the analyzed papers. When available, Ra had values ≤2 μm in samples prepared using airbrushing and radio frequency magnetron sputtering techniques [147,151,152]. Higher surface roughness with Ra around 10 μm was observed in wollastonite and wollastonite-diopsite glass-ceramic coatings deposited by thermal spraying [146] and in Sr-substituted bioactive glass coating applied by plasma spraying [148]. 

In Bellucci et al. [86], two different bioactive glass-ceramic coatings, 45S5 Bioglass and CaK, were deposited onto Ti-6Al-4V substrates using pulse electron deposition (PED) technique. An increase in the amount of crystallinity caused by the thermal sintering was found for both the coatings. CaK was less crystalline than 45S5 Bioglass before heat treatment, however, after annealing, the crystallinity of CaK samples increased to a higher extent, becoming much more crystalline than 45S5 Bioglass samples. As a consequence, the authors suggested that the increased roughness after sintering of 16.6% for 45S5 and 13.2% for CaK samples could be ascribed to an increase in crystallinity. In addition, as a consequence of an increase in roughness, an increase in water contact angle values were reported for both the coatings after annealing. This is a positive surface characteristic, because, contrary to hydrophobic ones, it has been widely demonstrated that hydrophilic surfaces can favour cell attachment, proliferation and differentiation [163].

Furthermore, in regard to the thickness, values between 1 and 200 μm were observed. The thinnest coatings (1–3 μm) were obtained by PED [86] and radio frequency magnetron sputtering [151,152]. The thickness of a bioactive glass coating produced using the conventional vitreous enameling technique was found to be around 70 and 100 μm [153]. In Sanyal et al. [147], Scanning Electron Microscopy (SEM) analysis on cross-sections of the glass-ceramic coated Ti-6Al-4V substrates revealed that the coating thickness was about 53 ± 10 μm, showing lower values as compared to those of bioceramic coatings deposited by airbrushing as well [132,133,134]. Both high velocity suspension flame spraying (HVSFS) and suspension plasma spraying (SPS) techniques allowed for the deposition of bioactive coating with a thickness below 50 μm [149].

Spraying techniques allowed for the obtainment of a wide range of coating thickness, from 30 to 200 μm [98,146,148,150]. 45S5 Bioglass coatings deposited onto Ti substrates—differing in thickness (41–83 μm) and porosity—were produced using the HVSFS technique modifying the parameter settings [150]. Pre-heating of the substrates was fundamental in reducing the rapid cooling of glass droplets, thus allowing the deposition of a first thin layer containing abundant fine porosity. The structure of the coatings showed a through-thickness microstructural gradient, with denser and thicker layers on the top thanks to the increasingly warm glass surface, which slowly cooled the impinging droplets. 

**Figure 2:**
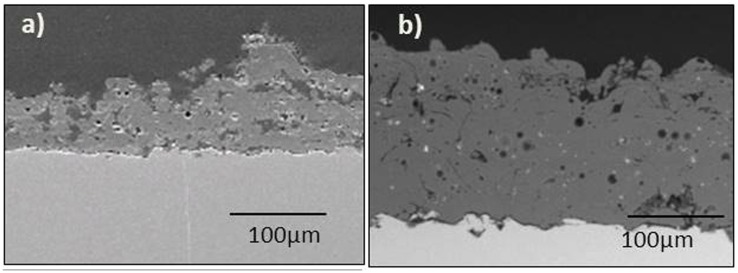
SEM micrographs of the cross section of: (**a**) wollastonite and (**b**) wollastonite-diopside glass ceramic coatings deposited on Ti-6Al-4V substrate, from Garcia et al., 2018 [146].

Wollastonite and wollastonite-diopside glass ceramic coatings, for the same number of torch passes, were characterized by a thickness of about 100–150 μm and 130–200 μm, respectively [146], as visible in Figure 2. Interestingly, the boundaries between adjacent layers associated to each torch pass were clearly detectable on SEM cross-sectional images. 

Bioactive glasses and silica-based ceramics coatings deposited onto Ti and Ti alloys have been largely investigated, whilst fewer works have focused on the development of coated stainless-steel substrates for biomedical applications. 

The coatings applied onto stainless-steel substrates analyzed in this review were mainly synthetized by the sol-gel technique and deposited by electrophoretic deposition (EPD) [155,156] or dip-coating [57,62,157]. On the other hand, Garcia et al. [146], deposited wollastonite and wollastonite-diopside coatings by thermal spraying. These authors are the only one who reported the surface roughness of the coatings. Wollastonite-diopside coatings deposited onto stainless steel substrates exhibited higher Ra and Rz (the mean peak to valley height) values than wollastonite ones, which might be due to the coarser size of the wollastonite-diopside feedstock powders. In addition, the authors suggested that the higher roughness of the grit blasted stainless steel substrates (Ra 5 ± 1 μm; Rz 44 ± 5 μm), as compared to that of grit blasted Ti-6Al-4V substrates (Ra 3.8 ± 0.2 μm; Rz 25 ± 2 μm), could have contributed to the final coating roughness, with 316L SS system achieving the highest values. Moreover, wollastonite-diopside coatings were found to be thicker and denser than wollastonite coatings. 

As demonstrated by SEM cross-section illustration of hardystonite coating on 316L SS, a crack-less coating of 14 μm was obtained using electrophoretic method [155].

Hybrid organic–inorganic coatings containing wollastonite [62,158] or bioactive glass [57] particles presented mean surface roughness of 1.1 and 4.2 μm, respectively. 

Besides non-resorbable metals, also resorbable metals such as Mg alloys were successfully coated with bioactive glass-ceramics or silica-based ceramics. Bioactive silicate ceramics were applied onto Mg alloys of both AZ and ZK groups, using several deposition techniques. In order to enhance the corrosion resistance of ZK60 Mg alloy substrates, a rough and porous coating composed of MgO and Mg_2_SiO_4_ was deposited using the micro-arc oxidation (MAO) technique [11]. Using the same deposition process, Yu et al. [158] fabricated coatings containing MgO, Mg_2_SiO_4_ and a small quantity of Mg_2_Si_2_O_6_. SEM analysis on cross-section identified a coating layer with an average thickness of about 10 μm, characterized by a denser inner part, acting as a barrier decelerating the degradation rate of the substrate in corrosive body fluid. 

Various coatings containing diopside, merwinite or a combination of diopside, bredigite and fluoridated hydroxyapatite were applied to AZ91 Mg alloy using a combination of EPD method with other methods [65,66,67]. Among these studies, the average roughness and thickness were reported was only reported in one [67]. The merwinite coating possessed a mean roughness of 7 ± 1 μm and a thickness of approximately 250 ± 20 μm.

Eventually, bioactive 45S5 glass–ceramic coatings, deposited onto Mg alloy AZ31 substrates using dip-coating technique, exhibited an average thickness of ~1 μm [159,160,161,162]. In Rau et al. [69], RKKP glass-ceramic coatings on Mg-Ca alloy were characterized by a coating thickness about 100 μm. Moreover, the surface was composed of agglomerates, with an average surface roughness (r.m.s.) of 295 ± 30 nm, and of fine-texture aggregates, with r.m.s. of 47 ± 4 nm.

## 5. Coating-Substrate Adhesion Strength

Obtaining high coating-substrate adhesion is the most important purpose of coating deposition.

Several methods have been applied to investigate the interface characteristics. Most of the studies analyzed the presence of gaps at the interface or of cracks, along with coating thickness, on cross-section SEM or Optical Microscope images [57,98,155]. 

Scratch tests are commonly performed to obtain information about the adhesion strength at the interface between metal implants and coatings. The normal and tangential loads of the scratch are normally reported as a function of distance; in addition, friction coefficient can be reported as well. After scratch tests, SEM or Optical Microscope images are helpful for the analysis of scratch lines and crack patterns. Moreover, EDS analysis can be performed on the scratched surface to better assess the tracks surface composition and the partial or complete removal of the coating material [86,98,132,133,147].

Other methods to investigate the adhesion of the coatings to the substrates included the tensile adhesion test according to ASTM D4541 [164] or ASTM C633--13 standards [165], in which the force at which the failure occurs and the characteristics of the failure are recorded in order to measure the bonding strength of the coating [153,164,165,166,167]. Moreover, nano-indentation tests on cross-sections are widely conducted to assess the interfacial strength between the coating and the metallic substrate [168]. Furthermore, the occurrence of delamination of the coatings can be recorded *in vivo* studies as well [169]. 

The adhesion of the coating to the substrate is widely investigated in literatures when a non-resorbable metallic implant is used. Whereas studies regarding resorbable metallic implants such as Mg and Mg alloys implants, are mainly focused on the coating effectiveness in delaying the degradation of the implants by means of dissolution testing and electrochemical corrosion. Main findings of the literature review are summarized in Table 5.

Among various bioceramics, hydroxyapatite Ca_10_(PO_4_)_6_(OH)_2_—a calcium phosphate ceramic—is likely the most thoroughly researched material for coating purposes due to its biocompatibility and similarity to natural bone [46,170]. Since the 1980s, plasma-sprayed HA coatings have been successfully deposited onto metallic implants, mainly Ti-based ones, in order to accelerate and improve the implant osseointegration. However, the prognosis of these implants remains controversial. Even positive long-term results in both load-bearing and non-load-bearing applications have been reported [171,172,173,174]. Poor bond strength of the HA layer to metallic implants has been documented as well [169]. The failure normally occurred at the interface between the coating and the implant, as confirmed by both mechanical and *in vivo* studies [169,175]. Recently, silicate substituted HA was developed with the main purpose of improving the HA bioactivity [176]. SiO_4_^4−^ substituted HA was deposited on CpTi substrates by RF Magnetron sputtering, giving coatings with a thickness less than one micron and a nano-sized structure. On one hand, the introduction of Si in HA reduced the elastic modulus and coatings with the Young modulus very similar to the substrates were obtained. On the other hand, scratch tests showed that the introduction of Si increased the material loss and penetration depth during scratch tests [177]. 

The mismatch of CTE, as in the case of HA, which possesses a CTE in the range of 14 (10^−6^ °C^−1^), and Ti-based substrates (see Table 1), is likely to be responsible of the delamination of the coating, determining the loosening of the implant [123,127]. Furthermore, the increased rate of delamination at the substrate-coating interface over time in clinical settings might reflect the continuous increase of the strength at coating-bone level during the healing phase [169].

The bonding strength required by the international standard (ISO 13779-2) [178] for bioactive HA coating implants is 15 MPa. When the bonding strength between the silicate bioceramic coating and the Ti-based substrate was measured in accordance with ASTM C-633, the mean values were always higher than 15 MPa [123,127,135,139,140,141,142,143,144,145]. 

In one study [138], the adhesion strength of both sphene and HA coatings on Ti-6Al-4V substrates was assessed by scratch test. The results revealed enhanced bonding strength of the sphene coating, compared with HA, with Hertz stress values of 17.4 ± 0.9 MPa and of 9.8 ± 0.6 MPa, respectively.

Furthermore, good adhesion of sphene (CaTiSiO_5_) coatings deposited by airbrushing onto CpTi was observed [132,133,134]. This might be due to the reduced difference between the coefficient of thermal expansion of CpTi and CaTiSiO_5_ compared to the one of CpTi and HA. 

Bellucci et al. [86] examined the adhesion of the coatings to the substrates using micro-scratch tests as well. A correlation between the mechanical resistance and the crystallinity was found. In particular, coatings of 45S5 before sintering exhibited a lower critical load (Lc) at which partial delamination of the coating occurred (Lc ~ 0.4 N); sintered 45S5 samples (Lc ~ 0.8 N) and as-deposited CaK samples (Lc ~ 1.1 N) showed similar intermediate values, while sintered CaK coatings resulted to be the most resistant (Lc 4.5 N). The authors underlined that high crystallinity is normally considered a drawback for bioglasses; however, sintered CaK glass showed promising properties for implant applications which should be further investigated. The new CaK composition may be promising for the production of implant coatings, with improved properties when compared to the well-documented 45S5 Bioglass.

SPS bioactive glass coatings on Ti-6Al-4V were developed and various sets of spray parameters were analyzed in order to determine the role of process parameters on coating features [98]. Amorphous coatings were successfully produced, with a limited crystallization of the glass leading to the formation of silicate-based secondary phases, i.e., wollastonite (CaSiO_3_) and Ca_2_SiO_4_. The mechanical properties of the samples resulted to be slightly worse than, for instance, those reported by Altomare et al. [150], who investigated the 45S5 Bioglass coatings deposited by HVSFS. However, the elastic modulus of BGCa1–BGCa5 coatings was similar to that of the cortical bone [98]. Therefore, as indicated by the authors, it might contribute in the reduction of the stress shielding effect. Moreover, all the coatings showed a good adhesion to the substrates, with critical load values in the range between 18 and 21 N. 

Regarding resorbable metallic substrates, the bonding strength of 45S5 glass–ceramic coatings deposited by dip-coating to Mg alloys was investigated using tensile adhesion tests [161,162].

In Niu et al. [161], the coated samples were thermally treated at 480 °C for 90 min under different pressures and the influence of the pressure on the bonding strength was analyzed. The minimum value was obtained when the treatment was performed without pressure (14.2 ± 2.0 MPa). Intermediate values were achieved with Ar pressure at 0.3 and 0.9 MPa, with bonding strength of 17.5 ± 1.9 MPa and 22.7 ± 2.2 MPa, respectively. The maximum value (26.8 ± 2.7 MPa) was obtained with pressure at 0.6 MPa.

In another work, the influence of the heat treatment temperature on the adhesion of the bioactive coating to the Mg alloy was investigated [162]. Increasing the temperature (from 300 to 500 °C), an improved cohesion strength of the coating was observed, consisting in a strong interface between the glass and the glass–ceramic particles. Meanwhile, a deterioration of the adhesion strength between the coating and the substrate occurred. Therefore, these opposite trends resulted in a maximum bonding strength when samples where heat treated at 450 °C (27.0 ± 2.9 MPa)—which is closed to what obtained by Niu et al. [161].

Si-HA coatings were deposited on Mg-based substrates. In these experiments, coatings of few micron thickness were obtained and showed a positive response to cell proliferation, due to a reduction of corrosion rate [179].

**Figure 3:**
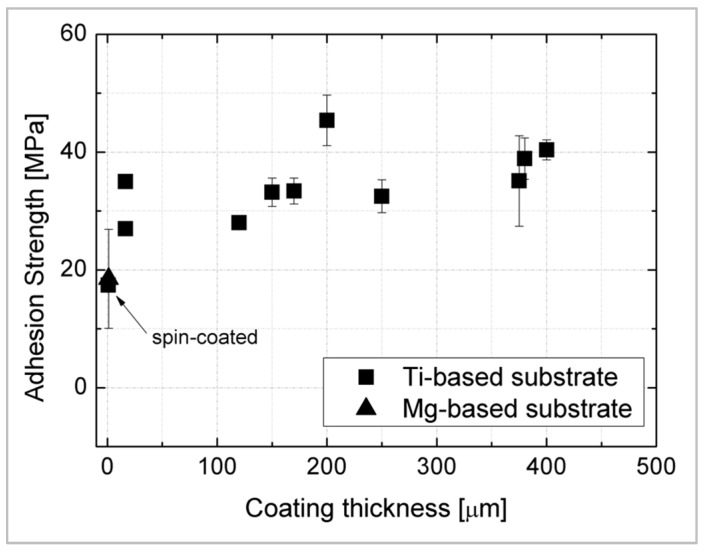
Adhesion strength and thickness of bioactive coatings. Data obtained by merging data available in Table 4 and Table 5.

In Figure 3, coating-substrate adhesion strength versus coating thickness is reported for those references where data are available. As it can be observed, adhesion strength for bioactive glass or bioceramic coatings on Ti-based substrates tends to slightly increase by increasing coating thickness when plasma spray is used as the deposition technique [123,127,139,140,141,142,143,144,145]. Only one datum is available for alternative techniques (i.e., spin-coating) [138] and one for coatings deposited on Mg-based substrates [162]—therefore no conclusion can be drawn.

However, it should be noted that the adhesion strength limit of 15 MPa defined for HA can be reached and overcome using alternative coatings compositions and deposition methods. A further limit for adhesion strength is that it should be tested *in ex-vivo* experiments, in order to evaluate the ideal adhesion strength value of the coatings once applied *in vivo* or in humans. 

## 6. Experiments in SBF Solution

In order to reduce the number of animal studies, various preliminary tests have been developed for screening the bioactive materials, such as the implant coating materials. The ability of a biomaterial to bond to living bone is often preliminarily assessed by investigating the ability of apatite to form on its surface in SBF [180,181,182]. When implanted *in vivo*, bioactive glasses were found to bond to the surrounding bone by forming a silica-rich layer and a calcium phosphate film on their surface. The capability to induce the formation of a calcium phosphate film *in vivo* was demonstrated to be reproducible in a cell-free solution (SBF), with ion concentrations similar to those of human blood plasma reproduced [180]. The original SBF was composted (in mM) of 142.0 Na^+^, 5.0 K^+^, 1.5 Mg^2+^, 2.5 Ca^2+^, 148.8 Cl^−^, 4.2 HCO_3_^−^, and 1.0 HPO_4_^2−^, but it lacked the SO_4_^2+^ ions contained in human blood plasma [183,184]. This composition was then corrected by Kokubo et al. by adding also SO_4_^2+^ ions [185,186].

In order to evaluate the *in vitro* apatite-mineralization ability, samples are soaked in SBF solution and analyzed at different time points. To characterize the morphological modifications of the surfaces and the formation of apatite crystals, various analyses can be performed, including SEM analyses occasionally combined with Energy Dispersive X-Ray Spectroscopy (EDS), surface roughness measurements, Fourier-transform infrared (FTIR) spectroscopy and X-ray diffraction (XRD) analysis. The variation of ion concentrations in SBF can be measured as well, using, for instance, Inductively Coupled Plasma Optical Emission Spectrometry (ICP-OES), Inductively Coupled Plasma Atomic Emission Spectrometry (ICP-AES) or Inductively Coupled Plasma Mass Spectrometry (ICP-MS). The concentration of different ions is not only determined before and after soaking at different time points, but the dissolution kinetics of ions can be obtained from the released concentrations as well.

The bioactivity of bioactive glasses, glass-ceramics and of silica-based ceramics deposited onto metallic substrates, was confirmed in several *in vitro* studies (see Table 6), thus indicating that these coatings might be suitable for the clinical applications improving the bonding of the metallic implantable devices with the surrounding bone tissue. 

All the studies reported in Table 6 focused on the surface analysis of the coating after soaking in SBF. Whereas, the chemical analysis of the solution was performed in only a few of the studies [66,141,143,144,146,147,150]. 

It is worth noting that most of the authors conducted the tests at different time points in order to monitor the modifications in the coating structure and the apatite-forming ability over time. The longest soaking time was 33 days [62,157]. In Wang et al. [53], seven days represented the longest immersion time, whereas in the other papers the maximum soaking time was at least double the time span.

Bioactive silicate ceramics possess a distinct apatite mineralization ability in SBF. In accordance with what reported by Wu and Chang [101], who indicated that wollastonite, dicalcium silicate, and bredigite, among the silica-based ceramics, had the best apatite forming abilities and fastest dissolutions in SBF. The apatite could be detected after one or two days of soaking, when Ti-based substrates coated with these bioceramics were immersed in SBF [53,143,144].

Interestingly, wollastonite samples, produced by means of atmosphere plasma spraying (APS) in combination with hydrothermal technology (HT), were found to possess a better bone-like apatite formation ability after the heat treatment, which could be due to their nano-sheet-like topography providing more sites for apatite nucleation [53]. 

A longer time for the induction of the apatite layer formation was reported for other coatings, such as akermanite [141], sphene [138], hardystonite [139], baghdadite [142] and diopside coatings [123]. However, it is to be noted that in some works the tests were performed after a minimum time of 14 days, so no data are available regarding the early phases after immersion [138,139,142].

Additionally, the bioactive glass and glass-ceramic coated Ti-based substrates were tested in SBF [1,98,146,147,149,150,154]. In Sanyal et al. [147], Ti-6Al-4V with a glass-ceramic coating, containing fluorapatite and diopside, showed a good bioactivity, as indicated by high concentration of Ca, P, F elements on the coating surface after soaking in SBF for 21 days. The formation of a fluroapatite layer was further confirmed by the elemental composition analyses of the solutions at different time points, showing an increase in Si and Mg concentrations and a decrease in Ca, P and F element concentrations with the increase in the soaking time.

**Figure 4:**
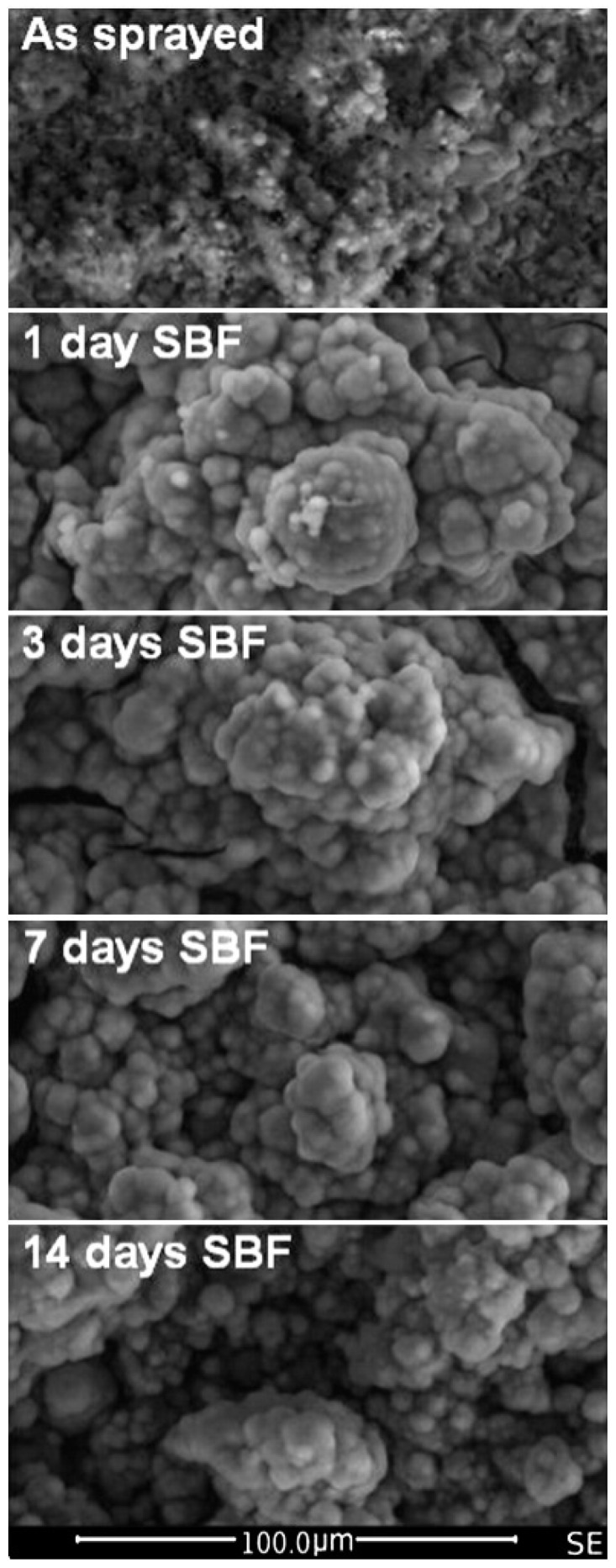
Surface evolution of the samples (BGCa3) immersed in SBF for increasing times, from Cattini et al., 2013 [98].

Encouraging results were obtained as well with suspension plasma sprayed bioactive glass coatings, composed of 4.7 Na_2_O, 42.3 CaO, 6.1 P_2_O_5_ and 46.9 SiO_2_ (in wt.%), produced using several sets of spray parameters and applied on rough Ti-6Al-4V disks (Ra = 3.4 μm) [98]. The degree of crystallinity and porosity of the different samples, which were mainly related to the spray distance, influenced the kinetic reaction in SBF—particularly in the first days of soaking. In fact, the crystalline phase (wollastonite) reacted with SBF, but at a lower rate as compared with the glassy one. It is worth noting, that, regardless of differences in the reaction rate among the samples, after 14 days of immersion all the surfaces were covered by a hydroxyapatite layer, replacing the gradually resorbed original coating, as shown in Figure 4.

Different *in vitro* behavior was noticed, when CpTi bioglass-coated samples were produced by different methods [149,150]. The bioactivity of 45S5 Bioglass coatings was characterized by soaking in SBF up to 28 days [150]. In order to verify whether the deposition process could affect the *in vitro* behavior of 45S5 Bioglass, bulk 45S5 Bioglass was tested as well. Coated samples and bulk bioglass presented the comparable interactions with SBF. After one day of immersion, a hydroxyapatite layer was distinguishable on all the samples, as confirmed by XRD patterns and micro-Raman spectra. At 28 days of soaking, the whole glass coating was replaced by the precipitated hydroxyapatite. At SEM analysis of the surfaces and on cross-sectional SEM micrographs, microcracks were detected, with larger ones on samples immersed in SBF for longer times. It is interesting to note that the samples did not release measurable amounts of Ti ions, as well as Al or Zr ions.

In Bolelli et al. [149], the apatite precipitation occurred on both HA and bioactive glass samples after soaking in SBF. Among the bioactive glass samples, the SPS coatings reacted more rapidly than the HVSFS coatings, due to their high porosity and to the related larger surface area in contact with the SBF—despite the poor mechanical properties exhibited by SPS bioactive glasses, which had already lost their mechanical integrity after one week of immersion in SBF. They could be useful for specific applications, such as for the release of functional bioactive polymers. Multilayered coatings could be designed as well, with a more stable inner layer deposited using the HVSFS technique and an outer SPS layer, in order to tailor the resorbability and reactivity of the coatings to specific need.

Furthermore, when the apatite forming ability of two bioactive glasses deposited onto Ti grade four substrates was compared to that of uncoated samples, better results were observed with the coated samples [1,154]. Positive *in vitro* results were obtained as well when 316L SS was used as a substrate.

In Bagherpour et al. [155], hardystonite coatings showed no change in their morphology after three days of soaking in SBF, while a cauliflower-shaped apatite was observed after one and two weeks of immersion. These observations further confirm the longer time required by hardystonite to induce the apatite formation, as compared to other biosilicates [101].

In Garcia et al. [146], it is not specified if 316L SS and/or Ti-based substrates were used for checking the *in vitro* bioactivity of the coatings. The attention was focused on the behavior of wollastonite and wollastonite-diopside glass-ceramic coatings in contact with the SBF. The incorporation of diopside was found to be a useful tool for adjusting the dissolution rate of wollastonite and, in the meantime, for enhancing the mechanical stability of the coating.

316L SS implants—characterized by a double-layer hybrid organic-inorganic sol-gel coating with the dispersion of wollastonite particles deposited using dip-coating technique—were able to induce hydroxyapatite deposition on their surface when immersed in SBF up to 33 days [62,157]. Consistently, hybrid organic-inorganic sol-gel coatings containing particles either of 45S5 Bioglass or of 45S5 glass (in which calcium was partially substituted strontium), exhibited hydroxyapatite deposition ability when immersed in SBF for 30 days [57].

Due to the rapid degradability of Mg and Mg alloys, a bioactive coating is expected to protect the substrate from the corrosive body fluid, thus favoring the maintenance of the mechanical integrity of the implants during the healing process. The *in vitro* behavior of a ceramic coating on Mg-based substrate—obtained by microarc oxidation using electrolyte containing silicate salts—was investigated by immersion in SBF for seven and 14 days. SEM-EDS analyses revealed the presence of apatite on the coating surface after seven days of soaking, while after 14 days a homogeneous bone-like apatite layer was detected on the surface, indicating a quick thickening of the apatite layer [158]. In a recent work of Razavi et al. [66], anodic spark deposition (ASD)—followed by EPD of an outer composite layer composed of diopside, bredigite, and fluoridated HA—was used to improve the properties of AZ91 Mg alloy. The coated samples resulted in moderating the degradation rate when immersed in SBF up to 28 days, as compared to AZ91 Mg alloy and AZ91 Mg alloy with only the intermediate layer deposited by ASD. SEM analysis revealed the presence of cracks on the surfaces as a consequence of the degradation—especially on uncoated AZ91 specimens. Moreover, composite/ASD/AZ91 samples showed a lower amount of weight loss and of Mg ion release into the solution, corroborating the protective effect of the coating against corrosion. Similarly, compression tests performed before and after soaking resulted in a less pronounced loss of mechanical integrity in composite/ASD/AZ91. The compressive strength of the samples decreased from 230 MPa, before soaking, to 100, 130 and 195 MPa for uncoated, ASD/AZ91, and composite/ASD/AZ91 specimens, respectively. 

Crack-free glass–ceramic coating, characterized by a glassy Si-based phase and a crystalline phase Na_2_Ca_2_Si_3_O_9_, acted as a protective barrier for the Mg alloy substrate, as confirmed by the immersion tests in SBF [159]. In accordance with these findings, Dou et al. [160] reported that optimized 45S5 glass–ceramic coatings produced onto Mg alloy through sol–gel and dip-coating method reduced the degradation of the substrate, improving the corrosion resistance in modified simulated body fluid. Although thinner coatings protected the substrates for degradation up to 3 days of soaking as well, a better protective effect over time was noticed with a thicker coating (~1 μm).

## 7. *In Vitro* Experiments

**Figure 5:**
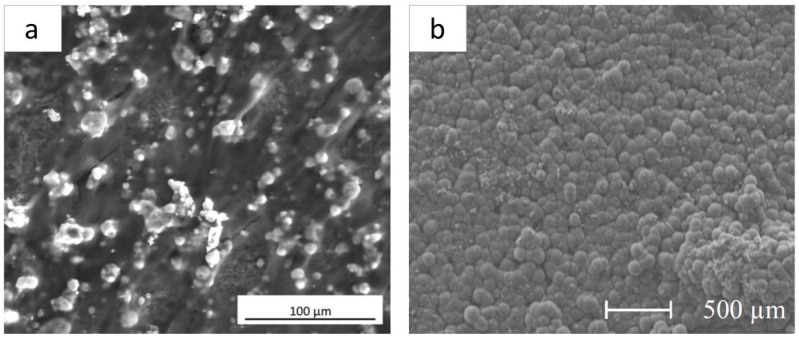
SEM images of cells grown on: (**a**) sphene-coated CpTi substrate after 21 days of culture in osteogenic differentiation medium; from Elsayed et al., 2018 [37]; (**b**) bioceramic coating composed of diopside, bredigite, and fluoridated hydroxyapatite deposited on Mg alloy (AZ91) after seven days of culture; modified from Razavi et al., 2018 [66].

*In vitro* tests, aiming to investigate the behavior of cells seeded onto implant surfaces, are considered a fundamental tool to preliminary determine the coating/tissue interaction on a cellular level. Among the studies analyzed in the present review (see Table 4), *in vitro* tests using different types of cells were performed to assess the biocompatibility, the osteoinductive properties and the antimicrobial ability of bioactive glass and glass-ceramic and silica-based ceramics (see Table 7). As revealed by SEM images, both Ti- and Mg-based coated substrates were found to sustain cellular growth *in vitro* (see Figure 5).

It is important to point out that most of the studies limited the investigation to cell viability by means of colorimetric assays, such as MTT (3-(4,5-dimethylthiazol-2-yl)-2,5-diphenyltetrazolium bromide) assay, MTS (3-(4,5-dimethylthiazol-2-yl)-5-(3-carboxymethoxyphenyl)-2-(4-sulfophenyl)-2H-tetrazolium) or WST-8 (2-(2-methoxy-4-nitrophenyl)-3-(4-nitrophenyl)-5-(2,4-disulfophenyl)-2H-tetrazolium sodium salt) assay.

### 7.1. In Vitro Behaviour of Ti-Based Substrates Coated by Bioactive Silica-BASED ceramics

Different silica-based ceramic coatings deposited onto Ti substrates, characterized by a rough surface with Ra in the range between 3.9 and 12.1 μm, were all able to support cell attachment and proliferation [37,127,135,139,140].

Regardless of the synthesis and deposition methods, sphene coatings were found to promote cell proliferation and differentiation [37,127,135]. Plasma-sprayed sphene-coated showed better results in terms of cell proliferation and alkaline phosphatase (ALP) activity than both plasma-sprayed HA coating and uncoated Ti-6Al-4V samples [127]. The authors speculated that the excellent cellular bioactivity might be due to the release of minor amounts of Ca and Si ions from the sphene coatings. This was in good agreement with the latter work [127], as well as with the results obtained by other researchers, who suggested that the dissolution of Ca and Si ions from sphene coatings might lead to beneficial effects by promoting cell proliferation and differentiation [37,135].

Furthermore, the cell proliferation rate on hardystonite coatings was found to be higher than on sphene coatings and uncoated Ti-6Al-4V substrates. The authors suggested that it could be caused by the release of zinc ions from the hardystonite coatings [135]. In accordance with the previous study, in Li et al. [139], cells were able to attach and spread on hardystonite coating surface, whereas fewer cells were counted on uncoated Ti-6Al-4V.

Moreover, comparable cell adhesion was observed on hardystonite and strontium-substituted hardystonite, in both cases superior than that on uncoated and HA-coated samples. Nevertheless, cell differentiation was superior on strontium-substituted hardystonite coatings compared to all the other groups. As both silica-based coatings had similar surface topography, it was suggested that the release of Sr ions—rather than surface topography—was responsible of the higher differentiation on strontium-substituted hardystonite compared to hardystonite ones [140,187,188].

Both akermanite and bredigite coatings stimulated the attachment and proliferation of bone marrow stem cells in a greater extent than HA coatings. Therefore, it is likely that the Mg and Si ions released from the coatings contributed to the improvement in cell proliferation *in vitro* [141,143]. 

Furthermore, calcium silicate coatings were fabricated by a combination of atmosphere plasma spraying and hydrothermal technology. The HT treatment allowed the glassy phase to be transformed into a nano-structured phase with high crystallinity. This not only significantly reduced the degradation rate of the coatings, but enhanced their biological properties as well—even though no significant differences in the concentration of Si ions in culture medium was observed between the two groups (i.e., HT treated and not) [53].

Beside morphological aspects, such as roughness and crystallite size, cell proliferation seems to be affected by the interaction with released ions from the coating, capable of promoting at different levels cell growth depending on their chemical characteristics. More detailed investigations should be necessary in order to clarify the role of the ions on cell growth, as well as the chemical characteristics in favor of cell proliferation.

### 7.2. In Vitro Behaviour of Bioactive Glass Coated Ti-Based Substrates

It would be interesting to investigate the role of surface roughness and coating adhesion to substrate on *in vitro* behavior of bioactive coatings. However, no data is available on bioactive glass coated titanium substrates.

Bioactive glasses deposited onto Ti-based substrates by means of different techniques were found to favour cell growing as compared to uncoated samples [1,150,154]. Bioactive calcium silicate glasses produced using sol-gel and dip-coating technique were found to improve the *in vitro* responses of the titanium substrates [1]. Similar findings were observed using bioactive calcium silicate glass coatings fabricated using the same synthesis and deposition techniques, but were characterized by the addition of limited amounts of Ag_2_O [15]. HVSFS-deposited 45S5 bioglass coatings presented no cytotoxicity [150]. Moreover, when human osteosarcoma cells were seeded onto coated and uncoated samples, cell viability increased overtime in the first week on both 45S5 Bioglass and control samples, with no statistical differences between the two groups.

### 7.3. In Vitro Behaviour of Mg-Based Substrates Coated by Bioactive Silica-Based Ceramics

Regarding resorbable metallic substrates coated by bioactive silica-based ceramics, various studies indicated that these coatings can enhance the *in vitro* responses of the implants compared to uncoated samples [11,65,66]. No data is available in these studies about coating roughness. Only in Lee et al. [11], it is underlined that the implant surface, which became rough and coarse with several pores after coating deposition, had a positive effect on osteoblast-like cell adhesion and proliferation. Moreover, the addition of gallic acid positively and adversely affect the growth of bone-like cell and fibroblast, respectively.

### 7.4. Antibacterial Properties of Bioactive Coatings

Biomaterial-associated infections are still common problems in both orthopedics and dentistry. Surface modifications of metallic substrates, including the deposition of coatings with antimicrobial properties, can prevent the occurrence of implant-related infections [189,190]. In two studies, the antibacterial properties of Ti-based substrates coated with hardystonite [139] and with a bioactive glass containing silver (Ag) [154] were investigated. In both the works, *Staphylococcus aureus*, a common pathogen causing implant-related infections [191], was used for antibacterial tests. The inhibitory effect of zinc ions on bacteria was confirmed, as demonstrated by the marked decrease of total colony forming unit (CFU) of *S. aureus* after 18 h of interaction with hardystonite-coated samples as comparted to Ti-6Al-4V substrates [139]. In Catauro et al. [154], the antibacterial effect of silver contained in the coatings was confirmed. In fact, a uniform layer of bacteria was detectable on uncoated substrates, while the number of bacteria decreased with the increase of Ag content in the coatings. Nevertheless, high percentages of silver were found to negatively affect coating biocompatibility, likely due to the nitrate ions released from the coating. Therefore, bioactive glass coatings with 0.14% of Ag_2_O resulted to be the best compromise in terms of both antimicrobial and biological properties. The incorporation of ions, such as Ag or Zn ions, in calcium silicate coatings could represent a valid tool to reduce implant-related infections. 

## 8. *In Vivo* Experiments

Utilization of animals needs to follow strict rules in terms of ethical issues, design and execution of the study as well as extrapolation of the results [192,193,194]. In an effort to minimize the number of *in vivo* animal studies, alternative methods for assessing the bioactivity and the biocompatibility of implantable devices, such as immersion in SBF, have been developed and extensively used in pre-clinical phases [180]. 

However, once the safety and the osteoconductive property of the material has been determined, further *in vivo* tests are required to confirm these findings before the clinical use in humans [192,193,194].

Since some bioactive glass-based and silicate ceramic coatings have shown promising *in vitro* properties, *in vivo* tests have been performed in several animal models to investigate the ability of the coatings to promote the new bone formation and neovascularization. Among the analyzed studies (see Table 4), *in vivo* studies were carried out in only 10 works. The main results are reported in Table 8. Briefly, bone-implant contact (BIC%), which represents the percentage of the implant surface in contact with the surrounding bone on a microscopic level, is one of the mostly used parameters to measure implant osseointegration. It can be noted that, when reported [137,140,152], higher values were achieved by implants with silicate-based and HA coatings as compared to uncoated ones. The beneficial effect of bioactive coatings was confirmed as well by the higher failure load registered during push-out tests with silicate-based bioactive coatings compared to control HA-coated and uncoated implants [137,140,148]. Moreover, silicate-based ceramic coatings were found to protect Mg substrates from fast corrosion, thus improving bone healing [65,67].

Six studies evaluated implants placed in the long bones (femur and tibia) of rats, [57,62,157] or rabbits [65,67,148], while in the remaining four studies implants were tested in larger animal models [137,140,151,152].

The inclusion of one or multiple control groups in the study design is considered of particular interest [192]. Concerning this aspect, in all studies apart from one [157] coated implants were compared to control implants. 

As good adhesion of the coating to the substrate is crucial for the success of the implants, it would be interesting to verify if mechanical test could predict the bonding of the coating once implanted *in vivo*. Unfortunately, in only one paper preliminary adhesion tests prior to *in vivo* phase are reported [140], so any comparison between the studies cannot be carried out.

**Figure 6:**
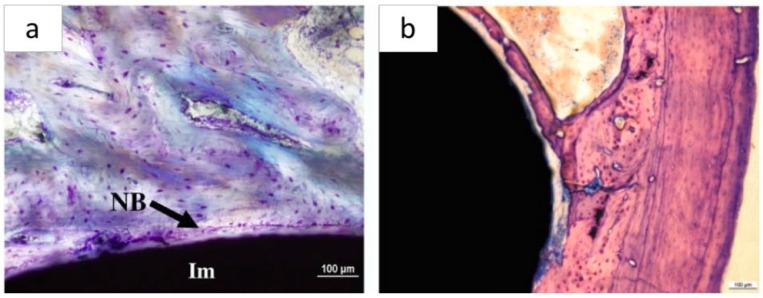
Histological morphology of the interface between the implant and the bone tissue of: (**a**) sphene-coated Ti-6Al-4V implant after six weeks of healing (Toluidine blue); from Ramaswamy et al., 2009 [137]; (**b**) stainless steel implant coated with hybrid organic-inorganic bioactive coating containing wollastonite after 60 days of healing (Toluidine blue); from Ballarre et al., 2011 [157]. Im, implant; NB, new bone.

As shown in Figure 6, metallic implants coated by bioactive coating were found to induce the new bone formation *in vivo*.

### 8.1. In Vivo Evaluation of Bioactive Coatings on Ti-Based Implants

HA has been extensively used as coating material for Ti implants. Interestingly, in the five studies analyzing the osseointegration of coated Ti-based implants, the test samples were always compared to HA-coated implants, while only three times to uncoated implants.

In Ramaswamy et al. [137], a similar bone to implant contact percentage was obtained by both sphene- and HA-coated Ti-6Al-4V implants. Contrary to uncoated samples, no fibrous tissue was detected around the coated implants, as indicated by the expression of ALP and by the presence of osteoclasts at the interface as well. Moreover, sphene-coated implants exhibited higher push-out values than uncoated implants, confirming the ability to strongly bond to the bone tissue.

Accordingly, in another study investigating silica-based ceramic coatings in a dog model, a better osseointegration was observed in presence of hardystonite and strontium-substituted hardystonite when compared to HA-coated and uncoated Ti implants [140]. It was demonstrated that strontium-substituted hardystonite-coated implants presented the highest osseointegration, followed in order by hardystonite-coated, HA-coated and, finally, uncoated implants.

The beneficial effect of strontium, in combination with a bioactive glass, was demonstrated as well. In Newman et al. [148], strontium-substituted bioactive glass-coated implants exhibited superior push-out strength than HA-coated controls, with a trend for increasing maximal shear strength over time. Interestingly, this is the only paper among the ones analyzed in the present review, in which three different implantation periods were used (i.e., six, 12 and 24 weeks). This characteristic was of help in the evaluation of this tendency. Moreover, it is to note that the majority of bioactive glass coating dissolved over a six-week time, thus stimulating bone formation around the implants.

The biological performances of Ti dental implants coated with composite HA/bioactive glass coatings were compared to those of HA-coated implants in both dog mandible [151] and goat iliac crest models [152].

When inserted in the healed sites in the mandible of Beagle dogs, implants with a high amount of bioactive glass (HABG_High_) showed significantly lower BIC% in comparison with both HA- and HABG_Low_-coated implants at four weeks post-operatively [151]. Nevertheless, after 12 weeks of healing, histomorphometical analysis revealed no significant differences between the experimental groups.

Furthermore, histological and histomorphometrical assessments, performed at four and 12 weeks after implantation in a goat iliac crest model, revealed that the incorporation of bioactive glass into the HA coating had significantly improved implant osseointegration as compared to uncoated implants [152]. However, similar results in terms of BIC% could be observed between HA- and HABG coated implants after 12 weeks of healing.

### 8.2. In Vivo Evaluation of Bioactive Coatings on Stainless Steel Implants

Regarding stainless steel substrates, hybrid organic–inorganic coatings containing wollastonite [62,157] or bioactive glass [57], produced by sol-gel technique and deposited by dip-coating, were tested in rat femur models.

*In vivo* osseointegration ability of hybrid organic–inorganic coatings functionalized with wollastonite was analyzed after 60 days of implantation. The newly formed bone around coated implants was characterized by osteoid and osteocyte lacunae, as well as by a laminar structure presenting osteoblasts at the interface [62,157]. In addition, in Ballarre et al. [62] a fibrous tissue encapsulating the uncoated control implants was observed. 

Consistently with these findings, the use of bioactive glass particles as a disperse phase in sol-gel protective coatings was found as well to enhance the bioactivity of stainless-steel implants [57]. An improved bone regeneration was documented for both kinds of coated implants as compared to uncoated ones. Nevertheless, coatings containing Sr-substituted bioactive glass were found to induce higher bone mineralization in the surrounding bone tissues than Bioglass 45S5-coated ones—in particular at the early phases after implantation.

### 8.3. In Vivo Evaluation of Bioactive Coatings on Mg-Based Implants

As previously mentioned, the main criticism of the use of Mg implants is their rapid degradability, leading to the early loss of the integrity of the implant and to undesired bubble formation. These two critical aspects were analyzed in both the animal studies on coated Mg alloys included in the present review. Resorbable Mg alloy implants coated with bioactive silica-based ceramics were tested in an experimental rabbit model [65,67].

The *in vivo* biocompatibility of double layer diopside/MAO coated AZ91 magnesium implants was examined in comparison with uncoated and MAO coated implants [65]. As mentioned above, one of the main drawbacks of Mg alloys is the formation of hydrogen gas due to the rapid corrosion of the implant [69]. Contrary to control implants, no bubble formation was visible on radiographs of diopside coated samples two weeks after surgery. As confirmed by histological analysis of two months post-op, the volume percentage of newly formed bone around diopside coated samples was approximately the double of that of control implants. Moreover, a limited implant weight loss of two months after implantation was observed in the test group compared to the control ones [65].

Using the same animal model, Razavi et al. [67] evaluated the biocompatibility of merwinite/PEO coating on the Mg alloy implants. As in the previous work, no gas bubbles were observed around implants with the bioceramic coating, whereas a few bubbles were detectable around PEO-coated samples and to a greater extent around uncoated implants. Additionally, the animals were sacrificed at two months post-operatively also in this work [67]. In accordance with the results reported in the previous study [65], the new bone volume percent was higher in the test group than in the control group. However, a lower amount of new bone formation was reported for merwinite/PEO coated implants (44%) than for diopside/MAO coated ones (65%). Similar to the previous study, implant weight loss at the end of the *in vivo* examination was higher in absence of bioceramic coating, thus confirming the role of silica-based ceramic coatings in protecting magnesium substrates from fast corrosion.

In the studies included in the present review, numerous silica-based bioactive coatings were applied. Despite the variety of animal models adopted and the numerous substrates and bioactive coatings utilized, a common trend towards an improved osseointegration of coated samples as compared to uncoated ones was observed in the majority of the studies.

## 9. Conclusions

Mechanical and *in vitro* biological analyses have demonstrated that bioactive glass-based and silicate ceramic coatings could be promising coating materials for orthopedic and dental applications. When optimized process parameters are used, new coatings deposited onto metallic implants seem to have appropriate adhesive properties to the substrates, overcoming the main drawbacks related to the use of previous generation of HA implants. Moreover, coatings deposited onto Mg alloy substrates were found to play a crucial role as protective barriers retarding the contact between the Mg alloy and the solution, thus favoring the maintenance of the mechanical integrity over a longer period of time. The bioactivity of the coatings has been investigated mainly by means of SBF immersion tests. Bioactive glass and silicate-based ceramic coatings showed sufficient efficiency of the apatite mineralization in SBF—higher than that of hydroxyapatite. The apatite forming ability and dissolution rate mainly depended on the chemical composition and structure of these coatings as well as the coating process, which controlled the total porosity and surface area of the coatings, in contact with the SBF. These parameters are useful tools for adjusting the coated implant performance in terms of dissolution rate, apatite formation and the mechanical stability of the coating. It is to be noted that available *in vivo* data on metallic implants coated with bioactive glass/glass-ceramics or silica-based ceramics are limited. Hence, it seems fundamental to expand the knowledge on the influence of chemical composition, mechanical properties and surface features of the coatings on implant performances *in vitro*, and—as well as in preclinical studies in animal models—in human clinical trials.

## Figures and Tables

**Table 1:** Different metallic materials and their mechanical, thermal and physical properties in comparison to bone. Data from CES EduPack™ 2018.

Metallic Material	E (GPa)	Hv (GPa)	CTE (10^−6^K^−1^)	Tm (°C)	*ρ* (Kg/m^3)^
CpTi	100–105	1.520–1.618	8.5–9.3	1670	4510–4520
Ti-6Al-4V	113–115	3.256–3.589 *	8.7–9.1 *	1610–1660	4430
Ti-6Al-7Nb	100–110	2.648–2.844 *	8–9.8	1530–1590	4510–4530
Ti-12Mo-6Zr-2Fe	63.1–90.1	3.207–3.383 *	8.7–8.89	1540–1620 *	4980–5000
316L SS	190–205	1.667–2.158	15–18	1380–1400	7870–8070
Mg	44–45.5	0.245–0.490	25.5–26.5	642–650	1730–1750
AZ91 Mg alloy	44–46	0.588–0.598	26–26.3	482–602	1800–1810
ZK61 Mg alloy	44–46	0.824–0.892 *	25–26 *	470–530 *	1803–1840
Bone	17–22	0.196–0.392 *	10–30 *	110–130 *^§^	1800–2100

* Estimated values; ^§^ glass Temperature; E = modulus of elasticity; Hv = hardness, Vickers; CTE = coefficient of linear thermal expansion; Tm = melting temperature; *ρ* = density.

**Table 2:** Common bioactive glasses (for example, but not limited) used in medical devices and their composition.

Commercial Name/Material	Composition (in wt.%)	Composition (in mol%)	References
45S5 Bioglass	45% SiO_2_,	46.1% SiO_2,_	[92,96,97]
	24.5% CaO,	26.9% CaO,	
	24.5% Na_2_O,	24.4% Na_2_O,	
	6.0% P_2_O_5_	2.6% P_2_O_5_	
S53P4	53% SiO_2_,	53.8% SiO_2_,	[92]
	20% CaO,	21.9% CaO,	
	23% Na_2_O,	22.7% Na_2_O,	
	4% P_2_O_5_	1.7% P_2_O_5_	
BG_Ca	46.9% SiO_2_,	47.2% SiO_2_,	[98]
	42.3% CaO,	45.6% CaO,	
	4.7% Na_2_O,	4.6% Na_2_O,	
	6.1% P_2_O_5_	2.6% P_2_O_5_	
CaK	46% SiO_2_,	47.2% SiO_2_,	[86]
	41% CaO,	45.6% CaO,	
	7% K_2_O,	4.6% K_2_O,	
	6% P_2_O_5_	2.6% P_2_O_5_	
13-93	53% SiO_2_,	54.6% SiO_2_,	[92,99]
	20% CaO,	22.1% CaO,	
	6% Na_2_O,	6% Na_2_O,	
	4% P_2_O_5_,	1.7% P_2_O_5_,	
	12% K_2_O,	7.9% K_2_O,	
	5% MgO	7.7% MgO	
Sr-Bioglass	41.5% SiO_2_,	44.5% SiO_2_,	[92]
	18.7% CaO,	21.5% CaO,	
	26.2% Na_2_O,	27.2% Na_2_O,	
	9.7% P_2_O_5_,	4.4% P_2_O_5_,	
	3.9% SrO	2.4% SrO	
70S30C	71.4% SiO_2_,	70% SiO_2_,	[92]
	28.6% CaO	30% CaO	
58 S	58% SiO_2_,	60% SiO_2_,	[87]
	33% CaO,	36% CaO,	
	9% P_2_O_5_	4% P_2_O_5_	
77 S	77% SiO_2_,	80% SiO_2_,	[87]
	14% CaO,	16% CaO,	
	9% P_2_O_5_	4% P_2_O_5_	

**Table 3:** Common silica-based ceramics used in medical devices and their composition, mechanical, thermal and physical properties (dense structure).

System	Materials	Compositions	CTE (10^−6^K^−1^)	E (GPa)	*ρ* (Kg/m^3^)	References
**Binary oxides**	Wollastonite	CaSiO_3_	10–13	52	2900	[108,109]
	Dicalcium silicate	Ca_2_SiO_4_	8.5	10–40	3150	[110,111]
	Tricalcium silicate	Ca_3_SiO_5_	_	24.9–36.7	3210	[112,113,114]
	Dimagnesium silicate	Mg_2_SiO_4_	_	_	3271	[115]
	Magnesium silicate	MgSiO_3_	_	_	2600–2800	[116]
	Zinc silicate	Zn_2_SiO_4_	_	_	3300	[116]
	Strontium silicate	SrSiO_3_	10.9	_	3650	[113,116]
**Ternary oxides**	Akermanite	Ca_2_MgSi_2_O_7_	9.9	42–56	2961	[117,118,119]
	Bredigite	Ca_7_MgSi_4_O_16_	_	43	3400	[120,121]
	Diopside	CaMgSi_2_O_6_	8.4	170	3200	[122,123]
	Merwinite	Ca_3_MgSi_2_O_8_	_	31–49	3150–3330	[119,124,125]
	Hardystonite	Ca_2_ZnSi_2_O_7_	11.2	37	3392	[118]
	Sphene	CaTiSiO_5_	6	_	3539	[126,127]
	Baghdadite	Ca_3_ZrSi_2_O_9_	_	82–120	3480	[128,129]

**Table 4:** Synthesis and deposition methods of bioactive coatings onto metallic substrates and their average roughness and thickness.

Substrate	Coating Material	Synthesis Method	Deposition Method	Coating Ra (μm)	Average Coating Thickness (μm)	References
CpTi	Sphene	Polymer-derived ceramics route	Airbrushing	4.1–6.5	50–100	[132]
CpTi	Sphene	Polymer-derived ceramics route	Airbrushing	3.1–8.4	133.8	[133]
CpTi	Sphene	Polymer-derived ceramics route	Airbrushing	0.5–1.4	120	[134]
CpTi	Sphene	Polymer-derived ceramics route	Airbrushing	3.9	–	[37]
Ti-6Al-4V	Sphene	Solid phase reaction	Plasma-spraying	7.5	–	[135]
	Hardystonite	Solid phase reaction	Plasma-spraying	7.5	–	
Ti	Sphene	Liquid phase reaction	Micro-arc oxidation	–	≤21	[136]
Ti-6Al-4V	Sphene	Sol-gel	Dip-coating	–	–	[137]
Ti-6Al-4V	Sphene	Sol-gel	Plasma-spraying	10	150	[127]
Ti-6Al-4V	Sphene	Sol-gel	Spin-coating	0.4	0.5–1	[138]
Ti-6Al-4V	Hardystonite	Sol-gel	Plasma-spraying	12.1	170	[139]
Ti-6Al-4V	Hardystonite	Solid phase reaction	Plasma-spraying	7.7	15–18	[140]
	Sr-substituted hardystonite	Solid phase reaction	Plasma-spraying	7.2	15–18	
Ti-6Al-4V	Akermanite	Sol-gel	Plasma-spraying	–	400	[141]
Ti-6Al-4V	Baghdadite	Solid state reaction	Plasma-spraying	9.8	120	[142]
Ti-6Al-4V	Bredigite	Sol-gel	Plasma-spraying	–	200	[143]
Ti-6Al-4V	Diopside	Commercially available powder	Plasma-spraying	8.3	200–300	[123]
Ti-6Al-4V	Dicalcium silicate	–	Plasma-spraying	–	380	[144]
Ti-6Al-4V	Wollastonite	Commercially available powder	Plasma-spraying	–	350–400	[145]
Ti-6Al-4V	Wollastonite	Liquid phase reaction	Atmosphere plasma spraying (+hydrothermal technology)	–	120–150	[53]
Ti-6Al-4V	Wollastonite glass-ceramic	Commercially available powder	Thermal spraying	9	100–150	[146]
	Wollastonite (36.77 in wt%)-diopside (63.23 in wt%) glass-ceramic	Commercially available powder (wollastonite); solid state reaction (diopside)	Thermal spraying	11	130–200	
Ti-6Al-4V	Bioactive glass-ceramic with glass phase (SiO_2_–Al_2_O_3_–CaO–P_2_O_5_–CaF_2_) and with fluorapatite (Ca_5_(PO_4_)_3_F) and diopside	Melting and crystallization	Airbrushing	0.4–1	53	[147]
Ti-6Al-4V	Bioactive glass in mol%: 23.41 SiO_2_, 3.18 CaCO_3_, 51.45 SrCO_3_, 8.67 MgO, 4.62 Na_2_CO_3_, 4.62 K_2_CO_3_, 3.47 ZnO, 5.20 Ca_3_(PO_4_)_2_	Melting	Plasma-spraying	11.9	50–100	[148]
Ti-6Al-4V	BG Ca	Melting	Plasma-spraying	–	30–40	[98]
CpTi	Bioactive glass (in mol%: 2.3 K_2_O, 2.3 Na_2_O, 45.6 CaO, 2.6 P_2_O_5_, 47.3 SiO_2_) + HA	Melting	High velocity suspension flame spraying	–	30	[149]
			Suspension plasma spraying	–	≤50	
Ti-6Al-4V	CaK	Melting	Pulsed electron deposition	–	1	[86]
	45S5 Bioglass	Melting	Pulsed electron deposition	–	1	
CpTi	45S5 Bioglass	Melting	High velocity suspension flame spraying	–	41–83	[150]
Ti	HA + Bioactive glass S53P4	Commercially available powder	Radio frequent magnetron sputtering	1.5–2	2–3	[151]
Ti	HA + Bioactive glass S53P4	Commercially available powder	Radio frequent magnetron sputtering	1.2	2.1	[152]
Ti-6Al-4V	Bioactive glass in wt.%: 59.1 SiO_2_, 19.2 CaO, 5.46 P_2_O_5_, 9.4 B_2_O_2_, 22.24 Na_2_O, 1.0 TiO_2_	Melting	Vitreous enameling technique	–	70–100	[153]
Ti grade 4	x CaO·(1−x)SiO_2_ bioactive glass (0.0 ≤ x ≤ 0.60)	Sol-gel	Dip-coating	–	–	[1]
Ti grade 4	70S30CxA bioactive glass (in mol%: 70 SiO_2_ (S), 30 CaO (C), x Ag_2_O (A), with 0.08 ≤ x ≤ 0.27)	Sol-gel	Dip-coating	–	–	[154]
316L SS	Hardystonite	Sol-gel	Electrophoretic deposition	–	14	[155]
316L SS	Hardystonite	Sol-gel	Electrophoretic deposition	–	–	[156]
316L SS	Wollastonite glass-ceramic	Commercially available powder	Thermal spraying	10	100–150	[146]
	Wollastonite (36.77 in wt.%)-diopside (63.23 in wt.%) glass-ceramic	Commercially available powder (wollastonite); solid state reaction (diopside)	Thermal spraying	13	130–200	
316L SS	Hybrid organic-inorganic + wollastonite	Sol-gel	Dip-coating	–	1.1	[62]
316L SS	Hybrid organic-inorganic + wollastonite	Sol-gel	Dip-coating	–	1.1	[157]
316L SS	Hybrid organic-inorganic + 45S5 Bioglass	Sol-gel	Dip-coating	–	4.2	[57]
	Hybrid organic-inorganic + 45S5 Bioglass with Ca partially substituted with 2mol% of Sr	Sol-gel	Dip-coating	–	4.2	
Mg alloy (AZ91)	Diopside + bredigite + fluoridated HA	Sol-gel	Anodic spark deposition + electrophoretic deposition	–	–	[66]
Mg alloy (AZ91)	Merwinite	Sol-gel	Plasma electrolytic oxidation + electrophoretic deposition	7	250	[67]
Mg alloy (AZ91)	Diopside	Sol-gel	Micro-arc oxidation + electrophoretic deposition	–	–	[65]
Mg alloy (ZK60)	Dimagnesium silicate–Magnesium oxide	Liquid phase reaction	Micro-arc oxidation	–	–	[11]
Mg alloy (ZK61)	Dimagnesium silicate + Magnesium oxide + Clinoenstatite	Liquid phase reaction	Micro-arc oxidation	–	10	[158]
Mg alloy (AZ31)	45S5 glass–ceramic	Sol-gel	Dip-coating	–	1	[159]
Mg alloy (AZ31)	45S5 glass–ceramic	Sol-gel	Dip-coating	–	0.5–1.0	[160]
Mg alloy (AZ31B)	45S5 glass–ceramic	Sol-gel	Dip-coating	–	–	[161]
Mg alloy (AZ31)	45S5 glass–ceramic	Sol-gel	Dip-coating	–	1.1	[162]
Mg-Ca (1.4 wt.%) alloy	RKKP *	Liquid phase reaction	Pulsed laser deposition	–	100	[69]

* RKKP: glass-ceramic material, RKKP stands for Ravaglioli A, Krajewski A, Kirsch M, Piancastelli A, a coating material with the following composition (in wt.%): 43.68 SiO_2_, 24 β-Ca_3_(PO_4_)_2_, 18.40 CaO, 4.92 CaF_2_, 4.53 Na_2_O, 2.78 MgO, 0.19 K_2_O, 1.00 Ta_2_O_5_, 0.50 La_2_O_3_.

**Table 5:** Coating-substrate adhesion strength.

Substrate	Coating Material	Test Performed	Adhesion Strength (MPa)	References
CpTi	Sphene	Scratch test	–	[132]
CpTi	Sphene	Scratch test	–	[133]
CpTi	Sphene	Scratch test	–	[134]
		Nanoindentation	–	
Ti-6Al-4V	Sphene	ASTM C-633	41.0 ± 3.5	[135]
	Hardystonite	ASTM C-633	27.0 ± 3.9	
Ti-6Al-4V	Sphene	ASTM C-633	33.2 ± 2.4	[127]
Ti-6Al-4V	Sphene	Scratch test	17.4 ± 0.9	[138]
Ti-6Al-4V	Hardystonite	ASTM C-633	33.4 ± 2.2	[139]
Ti-6Al-4V	Hardystonite	ASTM C-633	27 ± 4	[140]
	Sr-substituted hardystonite	ASTM C-633	35 ± 6	
Ti-6Al-4V	Akermanite	ASTM C-633	38.7–42.2	[141]
Ti-6Al-4V	Baghdadite	ASTM C-633	28 ± 4	[142]
Ti-6Al-4V	Bredigite	ASTM C-633	41.1–49.8	[143]
Ti-6Al-4V	Diopside	ASTM C-633	32.5 ± 2.8	[123]
Ti-6Al-4V	Dicalcium silicate	ASTM C-633	38.9 ± 3.5	[144]
Ti-6Al-4V	Wollastonite	ASTM C-633	27.4–42.8	[145]
Ti-6Al-4V	Wollastonite glass-ceramic	Microindentation test	–	[146]
	Wollastonite (36.77 in wt.%)-diopsite (63.23 in wt.%) glass-ceramic	Microindentation test	–	
Ti-6Al-4V	Bioactive glass-ceramic with glass phase (SiO_2_–Al_2_O_3_–CaO–P_2_O_5_–CaF_2_) and with fluorapatite and diopside	Scratch test	–	[147]
Ti-6Al-4V	BG_Ca	Scratch test	–	[98]
Ti-6Al-4V	CaK	Scratch test	–	[86]
	45S5 Bioglass	Scratch test	–	
316L SS	Wollastonite glass-ceramic	Microindentation test	–	[146]
	Wollastonite (36.77 in wt.%)-diopside (63.23 in wt.%) glass-ceramic	Microindentation test	–	
Mg alloy (AZ31B)	45S5 Glass–ceramic	Tensile adhesion test	14.2–26.8	[161]
Mg alloy (AZ31)	45S5 Glass–ceramic	Tensile adhesion test	10.1–27	[162]

**Table 6:** *In vitro* apatite forming ability of the coatings assessed by immersion SBF.

Substrate	Coating Material	Control	Soaking Time (days)	Surface Analysis	Ion Release Concentration	Main Results	Reference
Ti-6Al-4V	Sphene	–	21	SEM, EDS	–	Presence of nanocrystals of apatite on the surface.	[138]
Ti-6Al-4V	Hardystonite	–	28	SEM, EPMA	–	After 28 days, two layers were present on the coating surface: (a) Top layer: apatite layer, composed of Ca and P with a Ca/P molar ratio ~1.6. (b) Deeper layer: silica-rich layer, perhaps as a consequence of ionic exchange between Ca^2+^ in the coating and H^+^ in SBF.	[139]
Ti-6Al-4V	Akermanite	–	2, 6, 14	SEM, EDS, FTIR	ICP-OES	After two days: some apatite particles on the surface. After six days: thick layer of apatite. After 14 days: apatite layer (3 μm thick), silicon rich layer, original akermanite layer. High weight loss rate over the first six days, then, very low.	[141]
Ti-6Al-4V	Baghdadite	–	14, 28	SEM, EDS; XRD	–	Apatite formation already obvious after 14 days of immersion.	[142]
Ti-6Al-4V	Bredigite	–	2, 6, 14	SEM, EDS, FTIR, XRD	ICP-OES	Presence of apatite layer after two days, becoming denser after six days of soaking. After 14 days from outside to inner: apatite layer (thickness ~10 μm), silicon-rich layer and bredigite coating layer.	[143]
Ti-6Al-4V	Diopside	–	5, 15	SEM, EDS	–	After five days: isolated granular crystals composed of calcium and phosphorous. After 15 days: coating completely covered by apatite layer.	[123]
Ti-6Al-4V	Dicalcium silicate	–	2, 7, 14, 21	SEM, EDS, XRD	ICP-AES	After two days: a carbonate-containing HA layer was formed on the surface of coating, with the presence of an intermediate silica-rich layer. The thickness of carbonate-containing HA layer increased over time.	[144]
Ti-6Al-4V	Wollastonite	Calcium silicate coating (without HT)	1, 3, 7	SEM, EDS, XRD, FTIR	_	HT at 180 °C for 24 h enhanced apatite-mineralization ability of the coatings.	[53]
Ti-6Al-4V	Wollastonite glass-ceramic	_	7, 14	SEM, EDS	ICP-AES	Wollastonite glass-ceramic coating exhibited significantly higher dissolution rate than wollatonite-diopsite glass-ceramic coating.	[146]
	Wollastonite (36.77 in wt%)-diopside (63.23 in wt%) glass-ceramic						
Ti-6Al-4V	Bioactive glass-ceramic with glass phase (SiO^2^–Al^2^O^3^–CaO–P^2^O^5^–CaF^2^) and with fluorapatite and diopside	_	7, 14, 21	SEM, EDS	_	Formation of fluorapatite layer onto the coating surface. Si and Mg elements were significantly increased in the SBF solution with the increase in soaking time. Ca, P and F elements were instead decreased.	[147]
Ti-6Al-4V	BG Ca	–	1, 3, 7, 14	SEM, EDS, XRD, micro-Raman spectroscopy	–	All the coatings developed a surface layer of hydroxy-carbonated-apatite. The reaction kinetics were influenced by the coatings’ porosity and degree of crystallinity.	[98]
CpTi	Bioactive glass (in mol%: 2.3 K_2_O, 2.3 Na_2_O, 45.6 CaO, 2.6 P_2_O_5_, 47.3 SiO_2_) + HA	HA	1, 3, 7, 14	SEM, XRD, micro-Raman spectroscopy	_	Porous SPS bioactive glass coatings more rapidly dissolved in SBF, as compared to HVSFS bioactive glass coatings. SPS HA was more stable than HA HVSFS coating	[149]
CpTi	45S5 bioglass	Bulk glass	1, 3, 7, 14, 28	SEM, EDS, XRD, micro-Raman spectroscopy	ICP-OES	After one-day presence of HA layer on the sample surface. After 28 days the glass coating was replaced by precipitated HA film.	[150]
Ti grade 4	xCaO·(1−x)SiO_2_ bioactive glass (0.0 ≤ x ≤ 0.60)	Uncoated	7, 21	SEM, EDS	–	After seven days: uncoated samples showed fewer bone-like apatite globular grains in comparison to coated samples. Precipitate increased with the increased in exposure time to SBF.	[1]
Ti grade 4	70S30CxA bioactive glass (in mol%: 70 SiO_2_ (S), 30 CaO (C), x Ag_2_O (A), with 0.08 ≤ x ≤ 0.27)	Uncoated	21	SEM, EDS	–	Coated samples showed the surface covered by apatite globular crystals. Coated samples were more bioactive than uncoated ones.	[154]
316L SS	Hardystonite	–	3, 7, 14	SEM, EDS, XRD	–	After three days: no changes in coating morphology. After seven and 14 days: presence of cauliflower-shaped apatite on the surface. Iincreasing cracks by the time of immersion.	[155]
316L SS	Wollastonite glass-ceramic	–	7, 14	SEM, EDS	ICP-AES	Wollastonite glass-ceramic coating exhibited significantly higher dissolution rate than wollatonite-diopsite glass-ceramic coating.	[146]
	Wollastonite (36.77% in wt.%)-diopside (63.23% in wt.%) glass-ceramic						
316L SS	Hybrid organic-inorganic + wollastonite	–	5, 33	SEM, EDS, XRD	–	An apatite-like layer was observed on the surface, mainly composed of Ca and P.	[62]
316L SS	Hybrid organic-inorganic + wollastonite	–	5, 33	SEM, EDX	–	After five days: a Ca-P rich phase was detected in proximity to wollastonite particles. After 33 days: presence of numerous Ca-P rich compounds.	[157]
316L SS	Hybrid organic-inorganic + 45S5 Bioglass	a) Stainless steel; b) double layer of TMS	30	SEM, micro-Raman assays	–	Formation of HA on both test surfaces.	[57]
	Hybrid organic-inorganic + 45S5 Bioglass with Ca partially substituted with 2 mol% of Sr						
Mg alloy (AZ91)	Diopside + bredigite + fluoridated HA	a) Coated Mg alloy (ASD/AZ91); b) Mg alloy (AZ91)	3, 7, 14, 21, 28	SEM, EDS, FTIR	ICP	Amount of degradation and precipitates on the surface: composite/ASD/AZ91 > ASD/AZ91 > AZ91.	[66]
Mg alloy (ZK61)	Dimagnesium silicate + Magnesium oxide + Clinoenstatite	–	7, 14	SEM, EPMA, FTIR	–	Quick growing of the apatite layer.	[158]
Mg alloy (AZ31)	45S5 glass–ceramic	Uncoated	1, 7, 14	SEM, EDS	–	Enhanced corrosion resistance of coated sample over the first seven days. After 14 days of soaking, reduced corrosion resistance in the coated samples as well due to the cracking of the coating.	[159]
Mg alloy (AZ31)	45S5 glass–ceramic	Uncoated	1, 3, 5, 7	SEM, EDS	_	Samples with the thickest coating, 3A500, showed lower (2.31%) mass loss than A500 (72.71%), 2A500 (72.24%) and uncoated (78.04%) samples, along with a lower pH variation of m-SBF after seven days.	[160]

ASD = anodic spark deposition; EPMA = electron probe micro-analyzer; HT = hydrothermal treatment; TMS = TEOS (tetraethoxysilane)–MTES (methyltriethoxysilane)–SiO_2_.

**Table 7:** Bioactive coatings: *in vitro* experiments.

Substrate	Coating Material	Control	Cells	Test Performed	Main Results	References
CpTi	Sphene	Uncoated	hADSCs	- MTT assay - SEM analysis - Immunofluorescence - Alzarin Red S staining - rt-PCR	Sphene-based coating significantly better supported cell attachment and proliferation, than CpTi samples. When cells were seeded in the presence of osteogenic differentiation medium for 21 days, a significantly higher accumulation of calcium deposits on sphene coatings than on uncoated samples was observed.	[37]
Ti-6Al-4V	Sphene	Uncoated	Primary human osteoblasts	- SEM analysis - MTS assay - rt-PCR	After seven days of culture, cell proliferation rate on hardystonite coatings was higher when compared with those on sphene coatings and Ti-6Al-4V samples (p < 0.05). Both coatings were able to enhance the expression of bone-related genes.	[135]
	Hardystonite					
Ti-6Al-4V	Sphene	HA-coated (Uncoated)	Human osteoblast-like cells	- SEM analysis - MTS assay - ICP-AES - ALP activity	- After seven days of culture, significantly higher cell proliferation and ALP activity on sphene coatings than on HA-coated and uncoated substrates were observed (p < 0.05). After seven days of culture, no detectable levels of Ti ions and minor amounts of Ca and Si ions released from sphene coatings.	[127]
Ti-6Al-4V	Hardystonite	Uncoated	MC3T3-E1 cells (a mouse calvaria-derived osteoblast-like cell line)	- SEM analysis - MTS assay	Hardystonite showed no toxic effect on cells. After 24 h incubation, cells on hardystonite coating were more elongated, spread and confluent than on uncoated samples.	[139]
Ti-6Al-4V	Hardystonite	HA-coated	Canine BMMSCs	- Immunofluorescence - rt-PCR - ICP-OES - ALP activity - Calcium deposition assay	After 14 days of culture, the expression levels for BMP-2, ALP and osteocalcin cells cultured on strontium-substituted hardystonite coatings were the highest, followed by hardystonite and then by HA coatings.	[140]
	Sr-substituted hardystonite					
Ti-6Al-4V	Akermanite	HA-coated	Rabbit BMMSCs	- SEM analysis - MTT assay	After one day, cells on HA coating were similar in appearance to those on akermanite coating, but with fewer minor filopodia. After seven days of culture, more cells were detected on the akermanite coating than on the HA one. After one day of culture no significant differences in cell proliferation rate between the two groups; cells on the akermanite coatings showed a higher proliferation rate than that on HA coatings at both three and seven days of culture (p < 0.05 and p < 0.01, respectively).	[141]
Ti-6Al-4V	Bredigite	- HA-coated - Blank control	Rabbit BMMSCs	- SEM analysis - MTT assay	Cells cultured on bredigite coating for one day presented an elongated morphology and were firmly attached to the surface. After three days of culture, the bredigite coating presented numerous cells on its surface, characterized by a net-like morphology. After three and seven days of culture, cells on bredigite coating had a higher proliferation rate than that on HA coating and blank control.	[143]
Ti-6Al-4V	Wollastonite	–	Rat BMMSCs	- MTT assay - ICP-AES - Immunofluorescence - ALP activity - qRT-PCR	Cells seeded on the HT treated coatings presented higher cell viability and proliferation than untreated coatings at all time points (one, four and seven days) (p < 0.05). Quantitative results of ALP activity cells cultured on HT treated and untreated coatings revealed higher ALP activity on HT treated samples at all time points (four, seven and 10 days) (p < 0.05). HT treatment enhanced the expression of osteogenic genes and angiogenic factors.	[53]
CpTi	45S5 Bioglass	Uncoated	Human osteosarcoma cell line MG63	- MTT assay - SEM analysis	After 24 h of culture, cells spread over the coated surface. After seven days, it appeared covered by a cell layer. Coated samples supported an increasing cell viability overtime, similarly to uncoated samples.	[150]
Ti grade 4	x CaO·(1 − x)SiO_2_ bioactive glass (0.0 ≤ x ≤ 0.60)	Uncoated	NIH 3 T3 murine fibroblasts cells	- WST-8 assay	After 24 h of culture, the cells grown on uncoated samples showed lower viability than on all coated samples (p < 0.05). The best results were obtained with 0.3CaO·SiO_2_ and 0.4CaO·SiO_2_ coatings, which were homogeneous and crack-free, contrary to SiO_2_, 0.5CaO·SiO_2_ and 0.6CaO·SiO_2_ coatings.	[1]
Ti grade 4	70S30CxA bioactive glass (in mol%: 70% SiO_2_ (S), 30% CaO (C), x% Ag_2_O (A), with 0.08 ≤ x ≤ 0.27	Uncoated	NIH 3 T3 murine fibroblasts cells	- WST-8 assay	Higher percentage of viable cells on coated samples than on uncoated ones. The coating with the lower content of Ag resulted to be the most biocompatible.	[154]
Mg alloy (AZ91)	Diopside + bredigite + fluoridated HA	(a) Uncoated; (b) ASD coated	L-929 fibroblast cell line	- MTT assay - SEM analysis	Increase in cell viability from two to seven days of culture in all samples. At all time points (two, five and seven days) cell viability was as follows: diopside + bredigite + fluoridated HA coated > ASD coated > uncoated.	[66]
Mg alloy (AZ91)	Diopside	(a) Uncoated; (b) MAO coated	L-929 fibroblast cell line	- MTT assay	Cell viability of all samples increased with the culture time. At all time points (two, five and seven days) cell viability was as follows: diopside coated > MAO coated > uncoated. Diopside coated samples had significantly higher cell viability than that of uncoated samples at all time intervals (p < 0.05).	[65]
Mg alloy (ZK60)	Dimagnesium silicate – Magnesium oxide	Uncoated	Human osteoblast-like cells (MG63) and NIH 3 T3 murine fibroblasts cells	- CellTiter-96 cytotoxicity test - SEM analysis	Dimagnesium silicate-magnesium oxide coatings, with or without gallic acid, favored osteoblast-like cell proliferation.	[11]

ALP = alkaline phosphatase; BMMSCs = bone marrow mesenchymal stem cells; BMP = bone morphogenic protein; hADSCs = human adipose-derived stem cells; qRT-PCR = quantitative reverse transcription polymerase chain reaction; rt-PCR = real time polymerase chain reaction.

**Table 8:** Bioactive coatings: *in vivo* experiments.

Substrate	Coating Material	Study Model *	Number of Test Implants	Control Implants ^§^	Sacrifice (wks)	Assessments Method	BIC%	Main Results	References
Ti-6Al-4V	Sphene	Merino sheep (femur) (n = 10)	20	(a) Uncoated (n = 20) (b) HA-coated (n = 20)	6	- Histological analysis - Histomorphometric analysis - Push-out testing	In cortico-cancellous bone: sphene-coated ~75% - HA coated ~75%, uncoated ~15%. In cortical bone: sphene-coated ~75%, HA coated ~80%, uncoated ~62%.	In cortico-cancellous bone, significantly higher BIC% in sphene- and HA-coated implants, than in uncoated ones. Uncoated implants in corticocancellous site: fibrous tissue and lack of ALP and TRAP staining at the interface. Push-out tests: significantly higher failure load with sphene-coated implants compared to uncoated ones in cortical bone.	[137]
Ti-6Al-4V	Hardystonite	Beagle dog (femur) (n = 12)	12 + 12	(a) Uncoated (n = 12) (b) HA-coated (n = 12)	12	- Sequential fluorescent labeling - Micro-CT analysis - Push-out test - Histomorphometric analysis	Sr-substituted hardystonite 51.20 ± 9.08. hardystonite 36.97 ± 8.72, HA 27.72 ± 5.48, uncoated < 10.	BIC% of Sr-substituted hardystonite-coated implants was higher than those of hardystonite (p < 0.05) and HA (p < 0.01). Push-out test (loading rate of 5 mm/min): Sr-substituted hardystonite-coated implants possessed the highest failure load (388.84 ± 100.51 N).	[140]
	Sr-substituted hardystonite								
Ti-6Al-4V	Bioactive glass (SrBG)	New Zealand rabbit (femur and tibia) (n = 27)	54	HA-coated (n = 54)	6,12, 24	- Push-out test - SEM analysis - Histological analysis - Histomorphometric analysis	Quantified using Osteomeasure software (OsteoMetrics)	No significant differences in BIC% between the two groups at any time point. Push-out: significant difference in maximal shear strength at 24 weeks between the two groups (p = 0.028). Maximal shear strength increased over time in bioactive glass-coated samples, but no similar increase in the control group.	[148]
Ti	HA + Bioactive glass S53P4	Beagle dog (mandible) (n = 16)	16 (HABG_High_) + 16 (HABG_Low_)	HA-coated (n = 16)	4, 12	- Histological analysis - Histomorphometric analysis	At four weeks: HA 41.5 ± 19.7, HABG_Low_ 45.1 ± 19.3, HABG_High_ 29.7 ± 12.5. At 12 weeks: overall BIC% ranged from 40.5% to 31.1% with no significant differences between the groups.	After four weeks, in HABG_High_ group BIC% was lower than in the other groups (p < 0.05). After 12 weeks, no significant differences in overall BA%, BIC% and first BIC among the groups.	[151]
Ti	HA + Bioactive glass S53P4 (HABG)	Saanen goat (iliac crest) (n = 8)	32	(a) Uncoated (n = 32) (b) HA-coated (n = 32)	4	- Removal torque testing - Histological analysis - Histomorphometric analysis	Monocortical: uncoated 40.7 ± 13.2, HA-coated 44.8 ± 21.7, HABG-coated 54.2 ± 18.4. Bicortical: uncoated 57.5 ± 8.5, HA-coated 65.7 ± 11.3, HABG-coated 66.7 ± 11.5.	HABG-coated implants showed higher (p < 0.05) BIC% in both monocortical and bicortical implant placements in comparison with uncoated implants.	[152]
316L SS	Hybrid organic-inorganic + wollastonite	Hokkaido rat (femur) (n = 4)	Unclear	Uncoated (n unclear)	8.5	- Histological analysis - SAXS analysis	~60 coated	After 60 days, newly formed bone around coated implants and fibrous tissue around uncoated implants. Uniform mean thickness of Ca/P rich crystals in the new bone tissue (~2 nm).	[62]
316L SS	Hybrid organic-inorganic + wollastonite	Hokkaido rat (femur) (n = 4)	Unclear	–	8.5	- Surface analysis (SEM, EDS, AFM) - Histological analysis - Nanoindentation	–	After 60 days, newly formed bone around coated implant, characterized by the presence of osteocyte lacunae and laminar structure.	[157]
316L SS	Hybrid organic-inorganic + 45S5 Bioglass	Wistar–Hokkaido rat (femur) (n = 6)	Unclear	Uncoated (n unclear)	4, 8	- SEM analysis - Micro-Raman Spectroscopy	–	Thickness of newly formed bone: at eight weeks ~50 μm for all the samples, but at four weeks lower bone thickness around uncoated implants. At four weeks post-op, a better mineralized tissue in samples with Sr-substituted bioactive glass than in those with 45S5 Bioglass coating.	[57]
	Hybrid organic-inorganic + 45S5 Bioglass with Ca partially substituted with 2 mol% of Sr								
Mg alloy (AZ91)	Merwinite	Rabbit (femur: greatee trochanter) (n = 3)	1	(a) Uncoated (n = 1) (b) PEO-coated (n = 1)	8	- Blood tests - Radiographs - Histological analysis - Histomorphometric analysis - Measurement of implant weight loss	–	On two-wks post-op radiographs: uncoated samples showed higher gas formation than PEO-coated ones, while no gas on test samples. Two months post-op new bone volume: merwinite (44%) > PEO-coated (31%) > uncoated (27%). Two months post-op: weight loss for uncoated, PEO-coated and merwinite coated implants was 25, 16, and 5 mg/cm^2^, respectively.	[67]
Mg alloy (AZ91)	Diopside	Rabbit (femur: greatee trochanter) (n not specified)	Not specified	(a) Uncoated (n not specified) (b) MAO coated (n not specified)	8	- Blood tests - Radiographs - Histological analysis - Histomorphometric analysis - Measurement of implant weight loss	–	No gas formation was clinically observed in any group. On two- weeks post-op radiographs: uncoated samples showed higher gas formation than MAO-coated ones, no gas on test samples. Two months post-op, volume percentage of newly formed bone around implants: diopside coated (65%) > MAO-coated (31%) > uncoated (27%) samples. Two months post-op: the weight loss for uncoated, MAO-coated and diopside coated implants was 25, 16, and 7 mg/cm^2^, respectively.	[65]

* sample size (number of animals) into brackets; ^§^ number of control implants into brackets; BA%= peri-implant bone area percentage; BIC = bone-implant contact; PEO: plasma electrolytic oxidation; SAXS = angle X-ray scattering; TRAP = tartrate-resistant acid phosphatase.
